# Ontogeny of ependymoglial cells lining the third ventricle in mice

**DOI:** 10.3389/fendo.2022.1073759

**Published:** 2023-01-05

**Authors:** David Lopez-Rodriguez, Antoine Rohrbach, Marc Lanzillo, Manon Gervais, Sophie Croizier, Fanny Langlet

**Affiliations:** ^1^ Department of Biomedical Sciences, Faculty of Biology and Medicine, University of Lausanne, Lausanne, Switzerland; ^2^ Center for Integrative Genomics, Faculty of Biology and Medicine, University of Lausanne, Lausanne, Switzerland

**Keywords:** third ventricle, tanycytes, ependymal cells, hypothalamic development, scRNAseq, BrdU

## Abstract

**Introduction:**

During hypothalamic development, the germinative neuroepithelium gives birth to diverse neural cells that regulate numerous physiological functions in adulthood.

**Methods:**

Here, we studied the ontogeny of ependymal cells in the mouse mediobasal hypothalamus using the BrdU approach and publicly available single-cell RNAseq datasets.

**Results:**

We observed that while typical ependymal cells are mainly produced at E13, tanycyte birth depends on time and subtypes and lasts up to P8. Typical ependymocytes and β tanycytes are the first to arise at the top and bottom of the dorsoventral axis around E13, whereas α tanycytes emerge later in development, generating an outside-in dorsoventral gradient along the third ventricle. Additionally, α tanycyte generation displayed a rostral-to-caudal pattern. Finally, tanycytes mature progressively until they reach transcriptional maturity between P4 and P14.

**Discussion:**

Altogether, this data shows that ependyma generation differs in time and distribution, highlighting the heterogeneity of the third ventricle.

## Introduction

The mediobasal hypothalamus controls different physiological processes and behaviors essential for life, ranging from feeding (1) to reproduction (2, 3).

Organized around the third ventricle (3V), the mediobasal hypothalamus developed from the germinative neuroepithelium during embryonic development (4). The embryonic ventricular zone is mainly composed of radial glial cells. These neural stem cells are bipolar progenitors producing neurons and glia and serving as scaffolds for newborn neural cells to reach their final destination within the brain parenchyma (5, 6). Classically, the mantle layer forms upon three consecutive waves of neurogenesis, leading first to lateral, then medial, and finally periventricular zones, as illustrated by the genesis of melanin-concentrating hormone (MCH) neurons in the lateral hypothalamus (LHA) (7, 8) or other neurons in the ventromedial nucleus (VMH) (9, 10). Later, the ventricular zone evolves to give the ependymal layer (11).

Along the 3V in the mediobasal hypothalamus, the ependymal layer is characterized by the presence of heterogeneous ependymal cells. First, ciliated and cuboidal cells −typical in diverse brain regions− line the upper part of the 3V and participate in circulating cerebrospinal fluid (12). A second cell type, characterized by a long process and the presence of only one or two cilia, lines the lateral wall and the bottom of the 3V. These peculiar ependymal cells called tanycytes are considered reminiscent radial glial cells within the brain and are currently subdivided into four different subtypes, α1, α2, β1, and β2 (13, 14). Restricted to circumventricular organs in mammals (15), tanycytes play a role in numerous neuroendocrine functions such as glucose homeostasis, energy balance, and reproduction (16–18).

While hypothalamic neurogenesis from the mantle layer is well documented in rodents (19, 20), the ontogeny of the ependyma within the mediobasal hypothalamus is still limited (13, 19, 21–23). Here, we used BrdU incorporation to provide a spatiotemporal characterization of the mouse gliogenesis along the ependymal layer. In addition, neuron birthdate was analyzed in the arcuate (ARH), the ventromedial (VMH), and the dorsomedial (DMH) nuclei of the hypothalamus for a comparative perspective. We first confirmed that the ependyma is mainly generated once the neighboring neurons are produced. However, this generation is highly heterogeneous regarding the cell subtypes. Indeed, we determined different spatiotemporal gradients for the generation of the ependyma on the rostrocaudal and dorsoventral axis. Using publicly available scRNAseq datasets, we finally highlighted the transcriptional pseudotime developmental trajectories giving rise to mature tanycyte and typical ependymal cell populations.

## Materials and methods

### Animals

C57Bl/6J mice (initially obtained from Charles River) were used in this study. Male and female mice were put together around 5 pm, and the presence of a vaginal plug was checked on the following day (before 9 am). In this case, the time of conception was documented and considered as embryonic day 0 (E0). The day of birth was regarded as postnatal day 0 (P0). All animal procedures were performed at the University of Lausanne and were reviewed and approved by the Veterinary Office of Canton de Vaud.

### Bromodeoxyuridine injections

BrdU crystals (5-Bromo-2-deoxyuridine, BrdU, Roche Applied Science, # 10280879001) were dissolved in 0.07 M NaOH solution warmed to 65°C. Pregnant mice (from gestational day 9 to 18) and male pups (from P0 to P8) were given a single i.p. injection around 10 a.m. (50mg/kg).

*Tissue preparation.* 21 to 23 days after birth (P21-P23), BrdU-injected male mice were anesthetized with isoflurane and perfused transcardially with a 0.9% NaCl solution, followed by an ice-cold solution of 4% paraformaldehyde in 0.1 M phosphate buffer, pH 7.4. Brains were quickly removed, postfixed in the same fixative for two hours at 4°C, and immersed in 20% sucrose in 0.1M phosphate-buffered saline (PBS) at 4°C overnight. Brains were finally either embedded in ice-cold OCT medium (optimal cutting temperature embedding medium, Tissue Tek, Sakura) and frozen in liquid nitrogen-cooled isopentane (most of the postnatally injected animals) or directly frozen in crushed dry ice without OCT (all prenatally injected animals). The different groups (n=3 to 7 per group) were then called “E9”, “E10”, “P1”, …: these developmental time points correspond to the day of the single i.p. BrdU injection.

### Immunohistochemistry

Brains were cut using a cryostat into 25-μm-thick coronal sections and processed for immunohistochemistry as described previously (24). Briefly, the slide-mounted sections were 1) incubated in a boiling 10 mM Citrate Buffer solution, pH 6.0, for 12 minutes; 2) blocked for 1 hour using a solution containing 2% normal goat serum and 0.3% Triton X-100; 3) incubated overnight at 4°C with primary antibodies (**Table S1**) followed by two hours at room temperature with a cocktail of secondary Alexa Fluor-conjugated antibodies (1:500, Molecular Probes, Invitrogen, San Diego, CA) (**Table S2**); 4) mounted with DAPI Fluoromount-G (Southern Biotech; REF: 0100-20).

### Antibody characterization

All primary and secondary antibodies used are listed in **Supplementary Tables S1**, **S2**. The rabbit polyclonal antibody to BrdU (Bio-rad Cat#AHP2405, RRID: AB_2922993) recognizes the synthetic thymidine analog bromodeoxyuridine (BrdU). The chicken polyclonal antibody to VIM (Vimentin) (Millipore Cat# AB5733, RRID: AB_11212377) produced a pattern of staining associated with tanycytes, ependymal cells, and endothelial cells, similar to that described elsewhere in the literature (25). The mouse monoclonal antibody to NeuN (Neuron-specific nuclear protein) (Millipore Cat# MAB377, RRID: AB_2298772) produced a pattern of staining associated with neuronal cells, similar to that described elsewhere in the literature (26).

### Microscopic imaging

Pictures were acquired using a ZEISS Axio Imager.M2 microscope, equipped with ApoTome.2 and a Camera Axiocam 702 mono (Zeiss, Germany). Specific filter cubes were used for the visualization of green (Filter set 38 HE eGFP shift free (E) EX BP 470/40, BS FT 495, EM BP 525/50), red (Filter set 43 HE Cy 3 shift free (E) EX BP 550/25, BS FT 570, EM BP 605/70), and blue (Filter set 49 DAPI (E) EX G 365, BS FT 395, EM BP 445/50) fluorescence. Different magnifications were selected using a Zeiss x20 objective (Objective Plan-Apochromat 20x/0.8 M27 (FWD=0.55mm)) and a 63× oil immersion objective (Objective C Plan-Apochromat 63x/1.4 Oil DIC M27 (FWD=0.14mm)). To create photomontages, images were acquired using ZEN 2.3 pro software using Z-Stack and Tiles/Positions ZEN modules for each fluorophore sequentially. Quintuple-ApoTome frames were collected stepwise over a defined z-focus range corresponding to all visible fluorescence within the section. Multiple-plane frames were collected at a step of 0.3 µm while using the x63 objective (between 43 and 60 frames per image) and 1 µm while using the x20 objective (between 11 and 18 frames per image). Weak deconvolution was finally applied to images following the acquisition. All images were saved in.czi, processed to get maximal intensity projections, and finally exported in.tiff. for the processing steps (*i.e*., adjust brightness and contrast and merge channels) using Adobe Photoshop (Adobe Systems, San Jose, CA)).

### Data analysis

**Figure 1:**
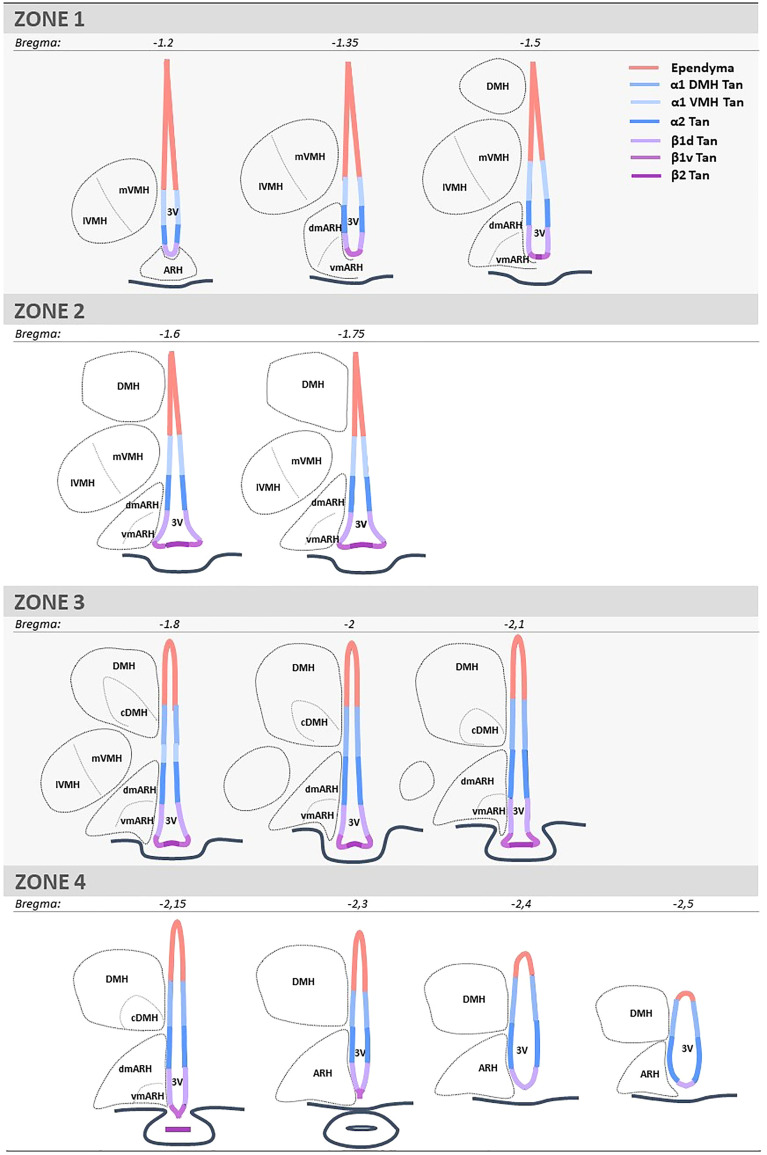
Schematic representation and coordinates of the four consecutive rostrocaudal zones used to analyze the mediobasal hypothalamus. Zone 1 corresponds to the anterior part of the ARH and the ME, where tanycyte processes are mainly found in the ARH and a few in the VMH. Zone 2 corresponds to the medial part of ME, where the bottom of the ventricle is more considerable, and tanycyte processes are found in both the ARH and VMH. Zone 3 corresponds to the medial-posterior part of the ME, where the VMH is lateral, and tanycyte processes are now observed in the DMH. Zone 4 corresponds to the posterior part of the ME and the presence of the infundibular stalk, where tanycyte processes are sent in the ARH and DMH. ARH is subdivided into vmARH and dmARH. VMH is subdivided into mVMH and lVMH. DMH is subdivided into cDMH. The ependyma is subdivided into 7 subregions: the ependymal layer facing the medial ME for beta-2 tanycytes (β2 Tan), the lateral ME for ventral beta-1 tanycytes (β1v Tan), the vmARH for dorsal beta-1 tanycytes (β1d Tan), the dmARH for alpha-2 tanycytes (α2 Tan), the VMH for rostral alpha-1 tanycytes (α1 VMH Tan), the cDMH for caudal alpha-1 tanycytes (α1 DMH Tan), and the dorsal part of the DMH for typical ependymal cells. 3V, third ventricle; ARH, arcuate nucleus of the hypothalamus; cDMH, compact dorsomedial nucleus of the hypothalamus; dmARH, dorsomedial arcuate nucleus of the hypothalamus; DMH, dorsomedial nucleus of the hypothalamus; lVMH, lateral ventromedial nucleus of the hypothalamus; ME, median eminence; mVMH, medial ventromedial nucleus of the hypothalamus; vmARH, ventromedial arcuate nucleus of the hypothalamus.

BrdU quantifications were performed using the AxioImager D1 microscope. Two investigators determined ependymocyte birthdate by counting the number of BrdU-positive and vimentin-positive cells. Tanycytes were differentiated from typical ependymal cells by their vimentin-positive basal process. One investigator quantified the neurogenesis by counting the number of BrdU-positive and NeuN-positive cells. The regions of interest (*i.e.*, the ventricular layer, the ARH, the VMH, and the DMH) were identified based on DAPI staining. For the ventrodorsal analysis, the ventricle was divided into seven subregions corresponding to the area where tanycyte processes are sent (*i.e.*, the medial median eminence, the lateral median eminence, the ventromedial ARH, the dorsomedial ARH, the VMH, and the DMH) and the layer composed of typical ependymal cells (**Figure 1**). For the rostrocaudal axes, the region was divided into four subregions, corresponding to zone 1 (from bregma -1.2 to -1.5mm), zone 2 (from bregma -1.6 to -1.75 mm), zone 3 (from bregma -1.8 to -2.1 mm), and zone 4 (from bregma -2.15 to -2.5 mm) (**Figure 1**). From a neuroanatomical point of view, these subdivisions were defined based on the shape of the ventricle and the presence/absence of hypothalamic nuclei along the 3V. These subdivisions were already described and used in former studies (27, 28).

Quantifications were performed on 3 to 7 animals per time point, on 5 to 12 sections per brain (1 to 3 sections per zone on average), and on both hemispheres. The total number of BrdU-positive cells was then calculated per zone and normalized by the number of analyzed sections (**Table S3**). The BrdU labeling pattern displayed low variability between animals injected at the same age.

### Single-cell RNAseq data analysis

Publicly available scRNAseq datasets were analyzed as described in their original papers with few modifications (26, 29). Developmental time points (E10-E16, E18, P4, P14, and P45) scRNAseq datasets from Kim et al. (26) (GSE132355) were independently analyzed using *Seurat 4.1.1* to identify the developmental age at which tanycyte-like cells can be found as an independent cluster. After generating a *SeuratObject* from raw data and splitting the matrix by developmental time points (“*orig.ident”*), we filtered cells to contain at least 200 features, and all datasets were normalized and scaled using *scTransform* (30). Following UMAP dimensional reduction (maintaining the first 50 PCA variables), we clustered cells using a resolution of 1.7 to maximize cluster separation. Furthermore, we used differential gene expression (DGE) analysis between clusters to identify major hypothalamic cell types using known cell marker genes from the original publication. Gene ontology analysis was performed using ShinyGO (v0.76) to obtain enriched GO terms using features expressed in tanycyte clusters at every developmental time point analyzed. ShinyGO was set to get the biological processes ordered by the false discovery rate (FDR) with a threshold of 0.05.

To identify the ependyma developmental trajectory, hypothalamic datasets from E11 to P45 (except P8) from Kim et al. (26) were integrated using *Harmony* (31). Normalization, dimensional reduction, and clustering were performed as described above. Following cell type identification, clusters containing progenitor cells (NPCs), ependyma, and tanycytes were subset in Seurat. The resulting Seurat object was then converted to *CellDataSet* to calculate the pseudotime trajectory from NPCs to the ependyma and tanycyte populations using Monocle3. A differentially expressed gene analysis across a single-cell trajectory from NPCs to ependyma and tanycyte populations was performed using the *graph_test* function.

To identify tanycyte subtypes across developmental time points, we analyzed the Yoo et al. (29) (GSE160378) scRNAseq dataset using Seurat. The original dataset was subset to contain the developmental time points P8 from wild-type mice exclusively. Data normalization, dimensional reduction, and cell-type identification were performed as described above. All analyses were performed in RStudio (v1.4.1103) using 4.1.1. version.

## Results

**Figure 2:**
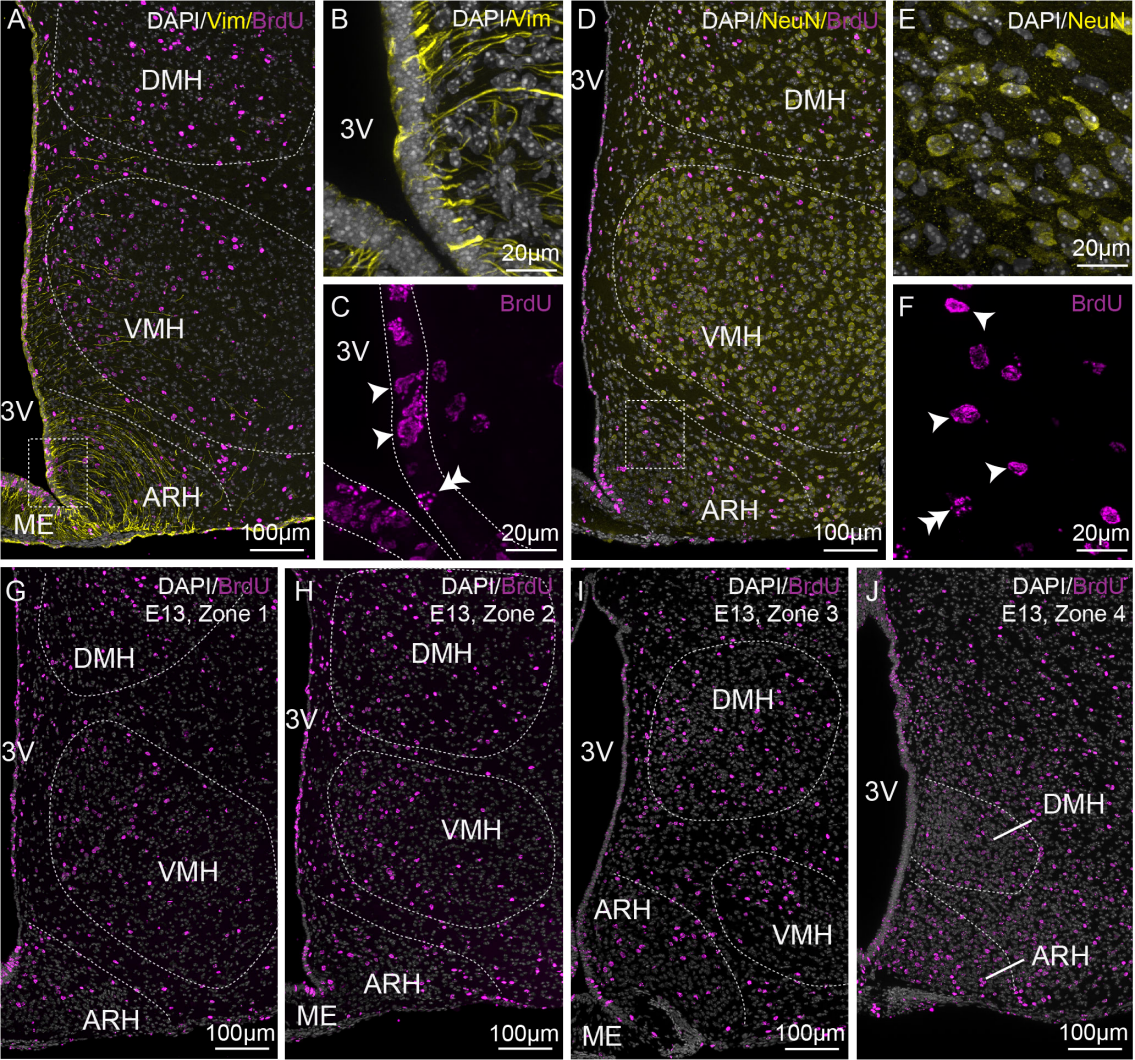
Characterization of BrdU-positive cells on the rostrocaudal axis. **(A–C)** Low- (x20, A) and high- (x63, B-C) magnification z-stack images showing the colocalization of vimentin immunoreactivity (yellow) and BrdU (pink) in zone 2 with Dapi counterstaining (white) in a coronal section from P21 male pups after BrdU injection to pregnant dams at E13. **(D–F)** Low- (x20, D) and high- (x63, E-F) magnification z-stack images showing the colocalization of NeuN immunoreactivity (yellow) and BrdU (pink) with Dapi counterstaining (white) in a coronal section at E13 in zone 2. **(G–J)** Low-magnification z-stack images (20x) showing the distribution of BrdU immunoreactivity (pink) with Dapi counterstaining (white) in coronal sections at E13 in zone 1 **(G)**, zone 2 **(H)**, zone 3 **(I)**, and zone 4 **(J)**. “E13” indicates the BrdU injection time point. Single arrowheads point out a BrdU labeling filling out the entire cell nucleus, whereas the double arrowheads point out a labeling that does not. ARH, arcuate nucleus of the hypothalamus; DMH, dorsomedial nucleus of the hypothalamus; ME, median eminence; VMH, ventromedial nucleus of the hypothalamus; 3V, third ventricle.

To analyze the birth of ependymal cells and neurons within the periventricular zone in the mediobasal hypothalamus, pregnant dams (from E9 to E18) and male pups (from P0 to P8) were given a single i.p. injection of BrdU, and its labeling was then performed on coronal brain sections from 21- to 23-day-old male mice. Vimentin (**Figures 2A–C**) and NeuN (**Figures 2D–F**) were co-stained to visualize ependymal cells and neurons, respectively. We first observed a differential pattern of BrdU labeling along the ventricle (**Figures 2A, C**). Indeed, at a younger age, BrdU is incorporated along the ventricle, but its labeling does not fill out the entire nucleus of the cell (**Figure 2C**, double arrowhead), suggesting additional cell divisions afterward and highlighting a high generative capacity. Alternatively, BrdU staining filled out the entire cell nucleus (**Figure 2C**, arrowheads), indicating no additional cell divisions and, consequently, the birth of the cell. For the analysis, we focused on full BrdU-staining in ependymal cells (**Figures 2A–C**) and neuronal cells (**Figures 2D–F**). The ependymal cells were further subdivided into typical cuboid ependymal cells *versus* tanycytes based on the presence of a basal vimentin-positive process (**Figures 2A, B**).

**Figure 3:**
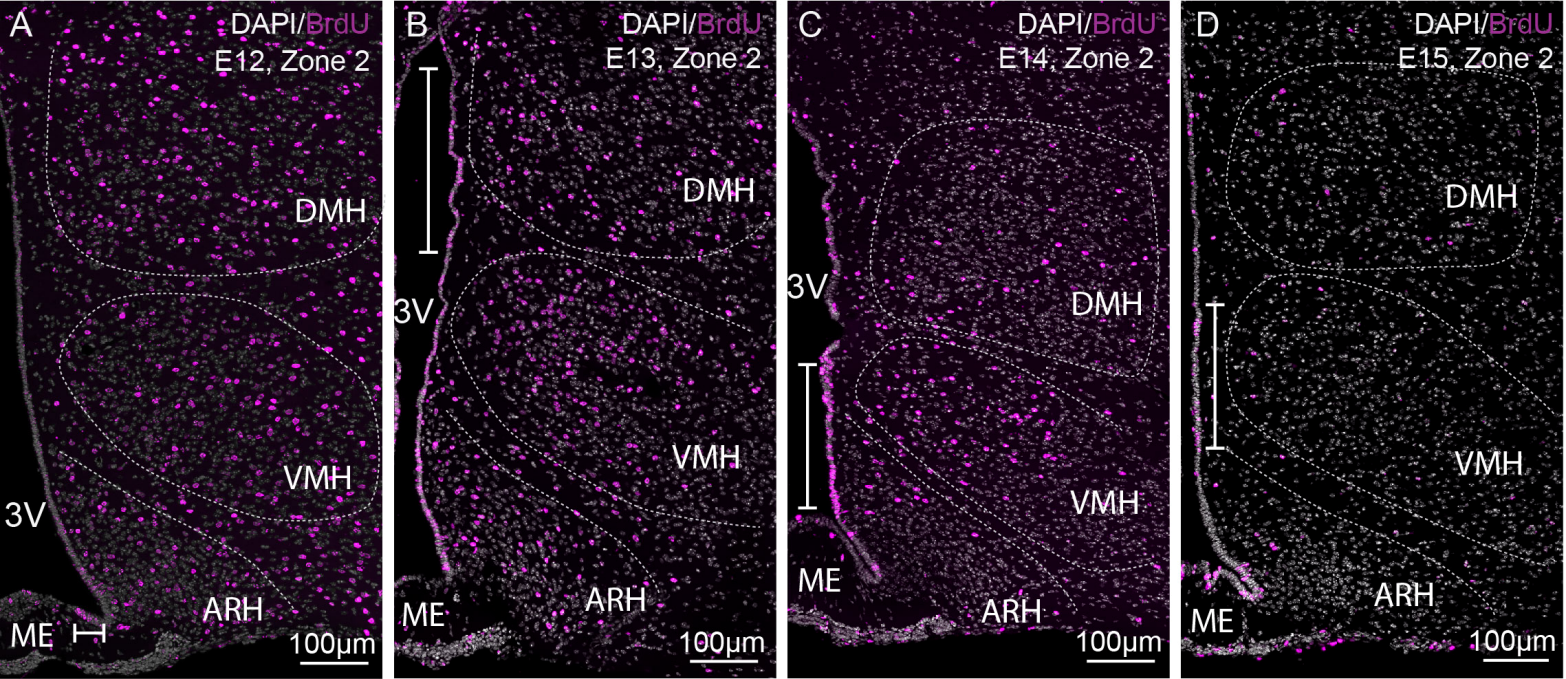
Characterization of BrdU-positive cells in time. **(A–D)** Low-magnification z-stack images (20x) showing the distribution of BrdU immunoreactivity (pink) in zone 2 (bregma -1.8) with Dapi counterstaining (white) in coronal sections from P21 male pups after BrdU injection to pregnant dams at E12 **(A)**, E13 **(B)**, E14 **(C)**, and E15 **(D)** (labeled “E12”, “E13”, “E14”, and “E15” on the pictures, respectively). The white bars point out the peaks of genesis along the third ventricle. ARH, arcuate nucleus of the hypothalamus; DMH, dorsomedial nucleus of the hypothalamus; ME, median eminence; VMH, ventromedial nucleus of the hypothalamus; 3V, third ventricle.

To adequately evaluate the heterogeneity of ependymoglial cell and neuronal birthdate along the 3V, a methodical analysis was performed on the ventrodorsal and rostrocaudal axes (**Figures 1**, **2G–J**). The 3V was first divided into seven subregions on the ventrodorsal axis: the ependymal layer facing the medial median eminence for β2 tanycytes, the lateral median eminence for ventral β1 tanycytes, the ventromedial ARH (vmARH) for dorsal β1 tanycytes, the dorsomedial ARH (dmARH) for α2 tanycytes, the VMH for rostral α1 tanycytes, the compact part of the DMH (cDMH) for caudal α1 tanycytes, and the dorsal part of the DMH for typical ependymal cells (**Figure 1**). Additionally, the region was divided into four subregions along the rostrocaudal axis, corresponding to zone 1 (from bregma -1.2 to -1.5 mm), zone 2 (from bregma -1.6 to -1.75 mm), zone 3 (from bregma -1.8 to -2.1 mm) and zone 4 (from bregma -2.15 to -2.5 mm) (**Figures 1**, **2G–J**). Finally, the analysis of newborn neural cells was based on a single BrdU injection performed at different time points from E9 to P8 to infer temporal gradients in BrdU labeling patterns (**Figure 3** and **File S1**). Thus, “E12” brains (*i.e.*, brains harvested from P21-22 male pups whose mothers received a single BrdU injection during pregnancy at E12) revealed a high generation of neurons and a low rate for ependymal cells at this time point (**Figure 3A**). In contrast, the ependyma mainly arises from E13 (**Figures 3B–D**) at different generation rhythms according to the ependymal subpopulations (**File S1**). Indeed, typical ependymal cells, α2 and α1 tanycytes display a higher generation at E13, E14 and E15, respectively (**Figures 3B–D**, brackets).

### Neuron birthdate in the mediobasal zone mostly occurs between E11 and E13 in mice

**Figure 4:**
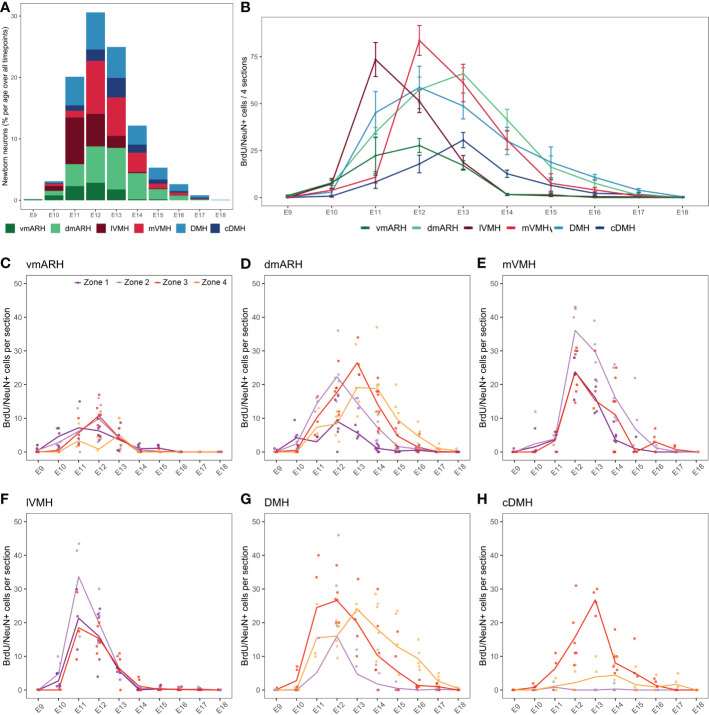
Developmental hypothalamic BrdU incorporation in NeuN-positive cells. **(A)** Stacked graph displaying the proportion of newborn BrdU/NeuN-positive cells in the hypothalamic regions vmARH, dmARH, lVMH, mVMH, cDMH, and DMH per age over all analyzed brains from P21-22 animals having received a single BrdU injection from E9 to P8 (*e.g.*, “E9” brains were harvested from P21-22 male pups whose mothers received a single BrdU injection during pregnancy at E9; “E10” brains were harvested from P21-22 male pups whose mothers received a single BrdU injection during pregnancy at E10…). The bars represent the percentage of cells per age over the whole analyzed period. Within a single age, the colors represent the percentage of cells per nucleus over the entire analyzed region. **(B)** Number (mean ± SEM) of BrdU/NeuN-positive cells per nucleus and per age over 4 hypothalamic sections (one in each rostrocaudal zone). **(C–H)** Number (average -line- and individual values -dots-) of BrdU/NeuN-positive cells per rostrocaudal zones (1 to 4) and per age in each hypothalamic region. n=3 to 7 animals per group. vmARH, ventromedial arcuate nucleus of the hypothalamus; dmARH, dorsomedial arcuate nucleus of the hypothalamus; lVMH, lateral ventromedial nucleus of the hypothalamus; mVMH, medial ventromedial nucleus of the hypothalamus; cDMH, compact dorsomedial nucleus of the hypothalamus; DMH, dorsomedial nucleus of the hypothalamus. See **Figure S1**.

In the periventricular (*i.e*., ARH and DMH) and medial nuclei (*i.e*., VMH), BrdU/NeuN-positive neurons were found in animals injected between E9 and E18 (**Figure 4A** and **Table S3**). No postnatal neurogenesis was observed. Thus, the neurogenic generative peak mainly occurs between E11 and E13, concentrating 75.6% of the total number of cells generated over the entire period (**Figure 4A**). However, BrdU/NeuN labeling patterns revealed regional differences (**Figures 4A, B**). First, most BrdU/NeuN cells were found in the dmARH, the DMH, the mVMH, and the lVMH with 24.0%, 22.7%, 20.9%, and 15.9% of generated cells over the entire developmental window (i.e., from E9 to P8), respectively (**Figures 4A, B** and **Table S3**). Lower generation levels were observed in the vmARH (8.2%) and the cDMH (8.3%) (**Figures 4A, B** and **Table S3**). Secondly, BrdU/NeuN labeling patterns confirmed a lateral-to-medial gradient in the generation of the VMH (9, 10). Indeed, most of the observed newborn neurons in the lateral VMH were generated at E11, whereas those within the medial VMH arose a day later at E12 (**Figure 4B** and **Figure S1**). Then, the neuronal generative peak in the periventricular nuclei (*i.e*., ARH and DMH regions) was concentrated within a few days between E12 and E13 without a lateral-to-medial gradient (**Figures 4B**, **S1**). Third, some nuclei additionally display a rostrocaudal gradient in their neuronal generation (**Figures 4C–H**). Specifically, the generation of newborn neurons in the vmARH, which mainly arises between E11 and E13 (**Figures 4B**, **S1**), actually starts in the rostral zone 1 at E10 and ends in the caudal region zone 4 by E13 (**Figure 4C**). In the dmARH, most neurons were born from E11 to E14 (**Figure 4B**), starting in the rostral zone 1 at E10 and ending in the caudal region zone 4 by E16 (**Figure 4D**). In contrast, VMH neurons were generated between E11 and E12 (**Figure 4B**) without a rostrocaudal gradient (**Figures 4E, F**). DMH neurons were generated between E11 and E14, with the first peak of genesis in zones 2 and 3 and a second in caudal zone 4 (**Figure 4G**). However, neuronal generation starts first and lasts longer in zone 3 and 4 (**Figure 4G**). In the cDMH, most neurons were generated in zone 3 at E13 (**Figure 4H**). To summarize, these different patterns of neurogenesis revealed a lateral-to-medial gradient for the VMH and a slight rostral-to-caudal direction for the periventricular ARH and DMH nuclei.

### The typical ependymal cell generation peaks at E13, whereas tanycytes are generated over an extended time window

**Figure 5:**
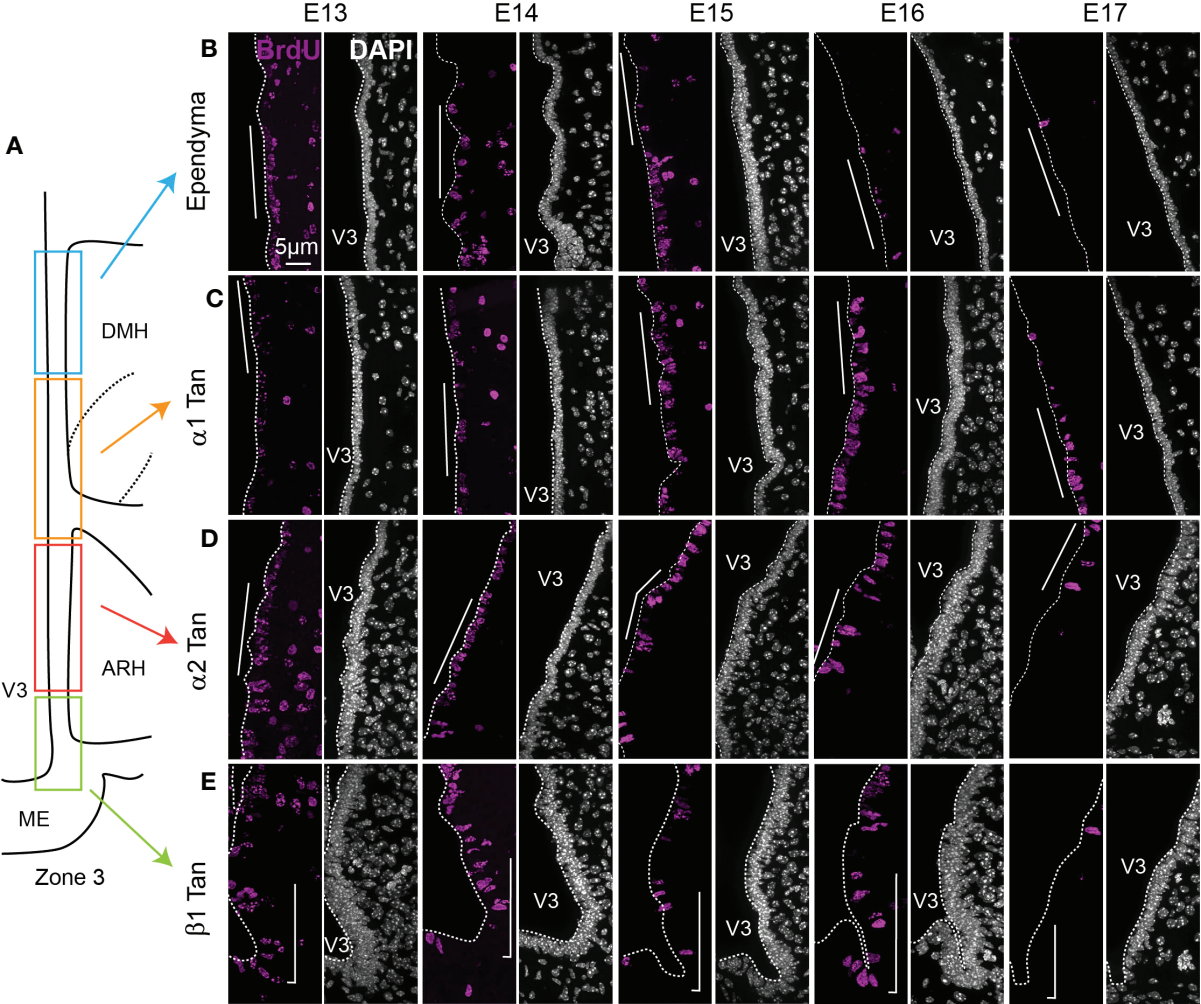
Characterization of BrdU-positive ependymoglial cells along the ventricular wall. **(A)** Schematic representation of zone 3 in the mediobasal hypothalamus. Rectangles display the different ependymal regions used for the analysis: beta-1 tanycytes facing the lateral ME and the vmARH (β1 Tan), alpha-2 tanycytes facing the dmARH (α2 Tan), alpha-1 tanycytes facing the cDMH (α1 Tan), and typical ependymal cells facing the dorsal part of the DMH. **(B–E)** High-magnification z-stack (63x) illustrative coronal images showing the distribution of BrdU immunoreactivity (pink) in the typical ependymal cells **(B)**, α1 **(C)**, α2 **(D)**, and β1 **(E)** from the developmental timepoint E13 to E17. DAPI counterstaining is represented in white. The white bars point out the respective cells of interest along the third ventricle. ARH, arcuate nucleus of the hypothalamus; DMH, dorsomedial nucleus of the hypothalamus; ME, median eminence; V3, third ventricle. The scale bar is shown in the figure. See **File S1**.

**Figure 6:**
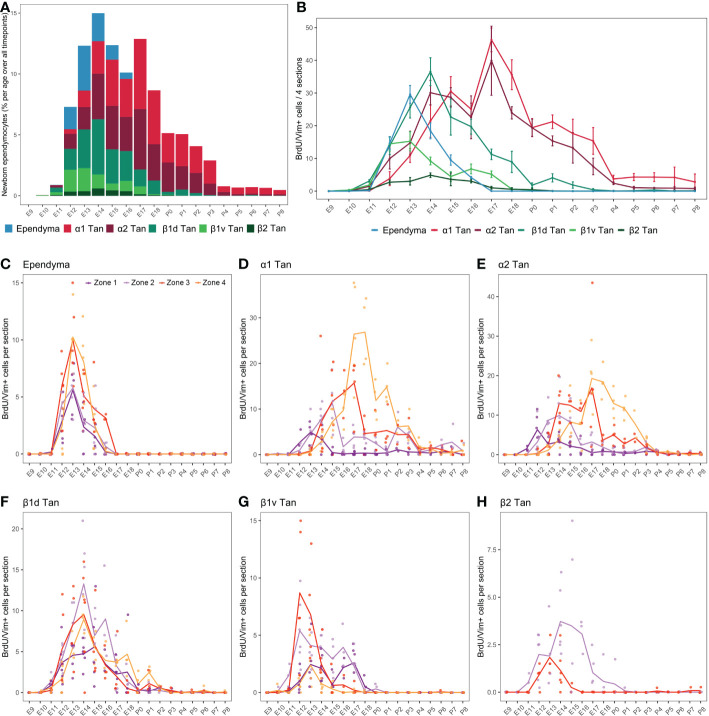
Developmental hypothalamic BrdU incorporation in vimentin (Vim)-positive cells. **(A)** Stacked graph displaying the proportion of newborn BrdU/Vim-positive cells in typical ependymal and tanycyte populations per age over all analyzed brains from P21-22 animals having received a single BrdU injection from E9 to P8 (*e.g.*, “E9” brains were harvested from P21-22 male pups whose mothers received a single BrdU injection during pregnancy at E9; “E10” brains were harvested from P21-22 male pups whose mothers received a single BrdU injection during pregnancy at E10…). The bars represent the percentage of cells per age over the whole analyzed period. Within a single age, the colors represent the percentage of cells per ependymal subpopulation over the whole analyzed region. **(B)** Number (mean ± SEM) of BrdU/Vim-positive cells per ependymal subpopulations and per age over 4 hypothalamic sections (one in each rostrocaudal zone). **(C–H)** Number (average -line- and individual values -dots-) of BrdU/Vim-positive cells per rostrocaudal zones (1 to 4) and per age in each ependymal subpopulation. n=3 to 7 animals per group. α1 Tan, alpha-1 tanycytes; α2 Tan, alpha-2 tanycytes; β1v Tan, ventral beta-1 tanycytes; β1d Tan, dorsal beta-1 tanycytes; β2 Tan, beta-2 tanycytes. See **Figure S2**.

Ependymal cell generation (*i.e.*, typical ependymal cells and tanycytes) occurred over an extended time window and appeared highly heterogeneous (**Figures 5**, **6** and **Table S3**). The distribution of full BrdU-positive cells along the 3V displayed different temporal, dorsoventral, and rostrocaudal gradients (**Figure 5** and **File S1**).

First, as observed for neurons (**Figure 4**), BrdU incorporation differed along the 3V (**Figure 6A**). While α tanycytes displayed high levels of generation, representing 62.0% (α1 = 33.0%, α2 = 29.0%) of newborn ependymal cells within the whole period (i.e., from E9 to P8), typical ependymal cells, β1, and especially β2 tanycytes, had low levels of generation, representing 9.5%, 26% (β1ventral = 7.3%, β1dorsal = 18.7%), and 2.5% of generated ependymal cells, respectively (**Figures 6A, B** and **Table S3**). Consequently, no clear generation peak was observed for β2 tanycytes, instead displaying a low generation between E11 and E18 (**Figure 6B**).

Across development, typical ependymal cells were generated in a short time window between E12 and E16, peaking at E13 with 38.5% of cell generation over the whole period (i.e., from E9 to P8) (**Figures 6A, B** and **Table S3**). In contrast, tanycytes were generated over an extended time window from E10 to P8 (**Figures 6A, B**). Specifically, we first found BrdU incorporation in β1 tanycytes occurring mostly between E11 and E18, with a peak at E12-13 for ventral β1 tanycytes and E14 for dorsal β1 tanycytes (**Figure 6B**). Next, α1 and α2 tanycytes arose with a more extended distribution throughout development, mainly between E12 and P3 (**Figure 6A**), with two generation peaks at E14-15 and E17 (**Figure 6B**). α2 tanycytes start to be generated one day before α1 tanycytes (**Figure 6B**). Therefore, these results show an “outside-in” generation pattern along the dorsoventral axis of the 3V. Indeed, cells were first generated in typical ependymal cells and β tanycytes in the dorsal and ventral regions between E12 and E14 and then in α tanycytes located in the central region between E14 and P3 (**Figure 6B**).

To further understand the extended time windows and the multiple peaks for the ependyma generation, we next analyzed BrdU incorporation along the rostrocaudal axis (**Figures 6C–H**; **S2**), revealing additional differences according to ependymal subpopulations. Ependymal cells were generated mainly at E13 in every zone without a rostrocaudal gradient (**Figure 6C**). Regarding α tanycytes, we observed a strong developmental rostral-to-caudal gradient in their generation. Indeed, α1 and α2 tanycytes are first generated in the rostral zones 1 and 2, followed by 3 and 4 later in development in a more prominent way (**Figures 6D, E**). This strong gradient explains the two peaks of generation observed at E14 and E17 when the rostrocaudal axis is not considered (**Figure 6B**). In contrast, β1 tanycytes facing the vmARH (β1d) did not display a clear rostrocaudal gradient of generation (**Figure 6F**). Interestingly, the generation of ventral β1 tanycytes (β1v) was more extended in zones 1 and 2 −from E11 to E18−, whereas it peaks at E12-13 in zones 3 and 4 (**Figure 6G**), suggesting a slight caudal-to-rostral gradient. Similarly, for β2 tanycytes, although we observed low BrdU incorporation in this cell subtype, the generation pattern suggests a slight caudal-to-rostral gradient with an extended generation up to P1 in zone 2 *versus* an earlier and shorter window in zone 3 (**Figure 6H**).

**Figure 7:**
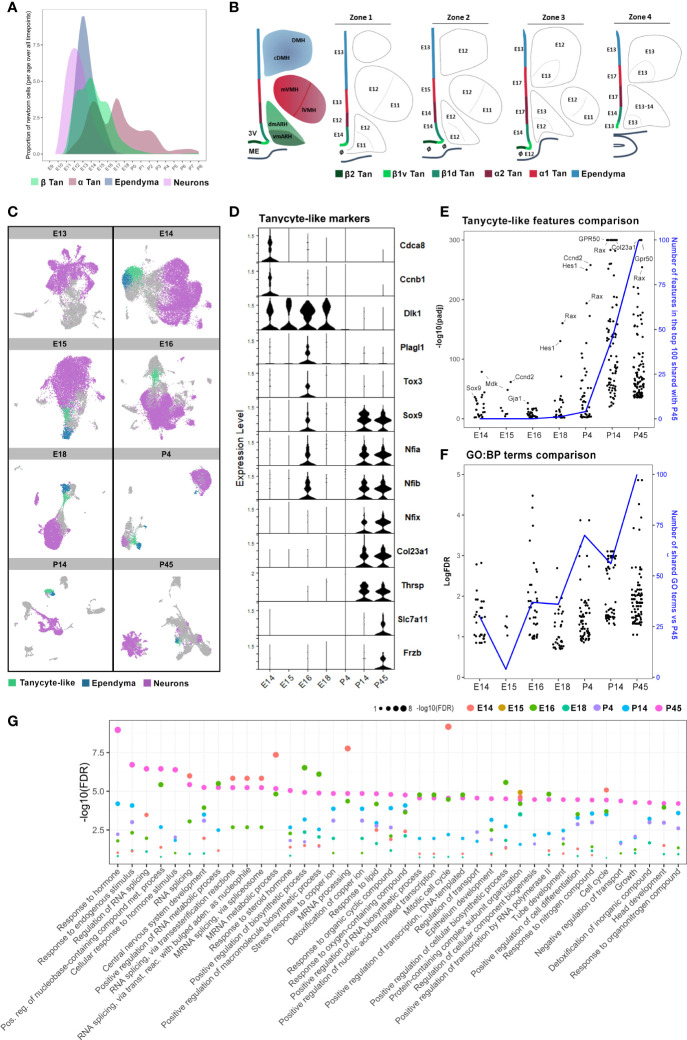
Cellular and molecular hypothalamic generation of mature tanycytes. **(A)** Representative plot illustrating the patterns of neuron, ependymal, and tanycyte generation across embryonic and early postnatal development extrapolated from our quantifications. **(B)** Rostrocaudal neuroanatomical representations of the developmental peaks of BrdU integration along the ventricular wall and hypothalamic nuclei. The ages indicate the peaks of genesis. The symbol ø indicates no clear peak of genesis (*i.e*., either multiple peaks or a continuous generation). **(C)** UMAP plots showing neuron (pink), ependymal (blue), and tanycyte-like (green) clusters in diencephalic and hypothalamic scRNAseq datasets (26), from E13 to P45, per developmental time point. **(D)** Violin plot showing specific features in tanycyte-like clusters across developmental time points from E14 to P45. **(E)** Comparison between the expression of the top 100 features of tanycyte-like cells at P45 *versus* those of tanycyte-like cells from every other developmental time point. The black dots represent features regarding their -log10(pAdj) significance. The blue line displays the number of features in the top 100 at each timepoint shared with the top 100 at P45. **(F)** Plot showing the comparison between the 100 more enriched GO terms from tanycyte-like cells at P45 (based on FDR) *versus* GO terms found in tanycyte-like cells from every other developmental time point. The black dots represent GO terms regarding their -log(FDR) significance. The blue line represents the number of GO terms shared in tanycyte-like clusters compared to P45. **(G)** Plot showing the -log10(FDR) of the top 40 more enriched GO terms in tanycyte-like cells at P45 compared to all time points. See **Figure S3**.

To summarize, our results demonstrated that typical ependymal cells (E13) and β tanycytes (E12-14) are the first generated shortly after the peak of neuron birthdate (E11-13) (**Figure 7A**). However, this time difference is less clear when considering the rostrocaudal gradient observed for neuronal generation (**Figure 7B**). Indeed, neurons are born before ependymal cells and β tanycytes in the rostral zone, but they arise concomitantly in the caudal region (**Figure 7B**). In contrast, there is little or no overlap between neuron and α tanycyte birthdate. Specifically, at the timing of the peak of neuron birth between E11 and E13, only a small proportion of α2 tanycytes is generated. α tanycytes arise later with a robust rostral-to-caudal gradient (**Figure 7B**).

### Tanycyte-like cells emerge as an independent cell population at E14 but are considered mature between P4 and P14

Our analysis revealed that neuron birthdate mainly peaks at E11-E13, ependymal cells at E13, and β and α tanycytes have a more widely distributed generation with multiple generative peaks between E12 and E18 (**Figure 7A**). To explore the developmental differences in the pattern of ependymal cell birthdate, we analyzed publicly available developmental hypothalamic scRNAseq datasets (26, 29). By using whole diencephalic or hypothalamic explants across embryonic and postnatal time points, Kim et al. (26) identified tanycytes to form an independent cluster starting at P4. Furthermore, they demonstrated that tanycytes diverge in trajectory from ependymal cells at E13 when they begin to express tanycyte and ependymal cell-specific markers, such as *Rax* and *Foxj1*, respectively (**Figure S3A**).

In agreement with their results, the analysis of the developmental scRNAseq datasets at each time point separately demonstrated that a population of tanycyte-like (*Rax*-positive) cells can be identified as an independent cluster starting from E14 (**Figure 7C**). Still, tanycyte-like cells do not share the same transcriptional profile across the different developmental time points. Indeed, DGE analysis of tanycyte-like cells at each developmental time point first showed that features commonly found in mature tanycytes, such as *Col23a1*, *Frzb, Slc7a11*, and *Thrsp*, are only observed in the P14 and P45 datasets (**Figure 7D** and **Table S4**). In contrast, genes involved in cell division, such as *Cdca8* and *Ccnb1*, are expressed specifically at E14 (**Figure 7D**). Similarly, tanycyte-like cells from embryonic time points express *Dlk1* and *Cdk4* up to E18 (**Figure 7D** and **Table S4**), consistent with their proliferative capacity during early development (6, 32). The cyclin-dependent kinase *Cdk4* is a master regulator of mitosis involved in cell proliferation (32), suggesting that these generating cells are likely radial glia. Interestingly, the genes *Sox9*, *Tox3*, *Plag1*, and several features from the NFI family of transcription factors (*Nfia, Nifb*), known to be involved in the negative regulation of neurogenesis (29) and the control of the onset of gliogenesis (33–35), are expressed at E16 (**Figure 7D**), corresponding to the end of neurogenesis peak and the burst in α tanycyte generation (**Figures 4**, **6**). Consistently, *Sox9* appears to be involved in the neurogenic-to-gliogenic fate switch (36). Secondly, we performed a comparative analysis of differentially expressed features in tanycyte-like clusters across development. Specifically, the 100 more significantly expressed genes in tanycyte-like cells at P45 were compared to tanycyte-like cell markers from the other developmental time points: most P14 and partially P4 tanycyte-like cells shared the same features compared to mature tanycytes at P45 (**Figure 7E**), highlighting the beginning of tanycyte maturation around P4. Indeed, we observed high levels of expression of the mature tanycyte markers *Col23a1*, *Gpr50*, and *Ccnd2*. Additionally, *Hes1*, a gene associated with the GO terms radial glia and neuroendocrine cell differentiation, shows an increase in expression levels starting from E18 (**Figure 7E**). Third, to further explore the immature *versus* mature state of tanycyte-like cells across developmental time points, we performed a gene ontology analysis focusing on biological process terms in tanycyte-like cells at P45 (**Figure 7F**) and compared the first 50 more significant GO terms to the other developmental time points (**Figures 7F, G**). Consistent with the known tanycyte functions (16–18), P45 GO terms comprise the response to hormones, endogenous stimulus, or lipids, and transport regulation (**Figure 7G**). As reported above, P4 and P14 tanycyte-like clusters shared the most GO terms compared to the older postnatal dataset (**Figure 7F**, blue line). Interestingly, many GO terms related to RNA processing and splicing are shared between E14 and P45 tanycyte-like clusters (**Figure 7G**). To summarize, these results suggest that while tanycytes-like cells are identified starting E14, these cells are not fully mature until early postnatal development, likely between P4 and P14.

Finally, to confirm the tanycyte maturity during postnatal development, we explored the occurrence of the different subtype specialization (*i.e.*, α1, α2, β1, and β2 tanycytes) (**Figures S3B, C**). Due to the high heterogeneity of cell types found in the hypothalamic dataset of Kim et al. (26) and the relatively low number of tanycytes identified per age, tanycyte subtypes are not distinguishable at any time point. To overcome this issue, we analyzed a scRNAseq dataset of *Rax*+ hypothalamic cells in wild-type mice at P8 (29). In agreement with the original article, our analysis allowed the identification of the tanycyte α1, α2, β1, and β2 cell populations (**Figures S3B, C**), suggesting that tanycyte subtypes are already present during early postnatal development.

### Transcriptional switch from gliogenesis to tanycyte maturation

**Figure 8:**
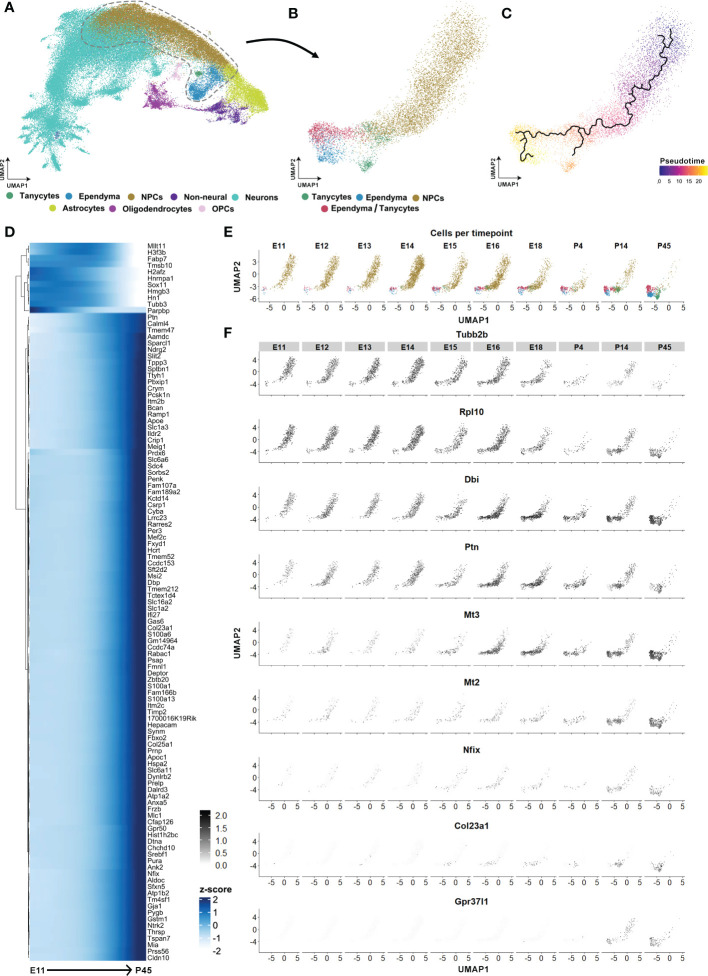
Tanycyte and ependyma developmental trajectories. **(A)** UMAP showing the integrated developmental scRNAseq datasets from hypothalamic and diencephalic explants between E11 and P45 (excluding E10 and P8 datasets) from Kim et al. (26). **(B)** UMAP showing the subclustering of progenitor cells (NPCs), tanycytes, ependyma, and tanycyte/ependymal cell populations. **(C)** UMAP plot displaying the pseudotime ependymal and tanycyte trajectories originating from NPCs. **(D)** Heatmap displaying the first 100 genes expressed in the pseudotime NPC to tanycyte trajectory. **(E)** UMAP displaying the development of tanycytes and ependymal cells split by age from E11 to P45. **(F)** UMAP showing features expressed across the developmental trajectory from NPCs to the tanycyte population. See **Figure S4**.

To explore the tanycyte maturation process, we finally performed an integrative analysis of the hypothalamic developmental dataset of Kim et al. (26), focusing on the developmental trajectory of tanycytes from undifferentiated cells. Following cell type identification, we subset the progenitor (NPCs), tanycytes, and ependymal cell populations (**Figures 8A, B**) and perform pseudotime trajectory analysis (**Figure 8C**). The pseudotime analysis allowed us to visualize the transitional state and the timing of the developing trajectory from NPCs to tanycytes *versus* typical ependymal cells, respectively (**Figure 8C**). Developmental trajectories for the tanycyte population identified an age around E16 at which there is a first switch in cell fate towards glia differentiation (**Figures 8D–F**). From E11 to E16, the genes *Tubb2b* and *Rlp10* involved in neurogenesis are highly expressed in the NPC clusters and rapidly decrease afterward (**Figure 8F**). At this time point (E16), the expression of genes involved in glial cell proliferation and differentiation (*i.e.*, *Dbi, Ptn, Nfix*) and the negative regulation of neurogenesis (*i.e., Mt3*) increases (**Figure 8F**). Furthermore, we observed starting at E16 but more clearly around P4-P14 that a second switch towards tanycyte maturation characterized by an increased expression tanycyte specific markers (*i.e.*, *Col23a1*) and genes involved in the negative regulation of astrocyte differentiation (*i.e.*, *Gpr37l1, Mt2)* (**Figure 8F** and **Table S5**). Finally, similar maturation patterns were found in the ependyma trajectory (**Figure S4** and **Table S5**). Specifically, starting from E16-18 and more clearly during the postnatal time points, ependyma begins to express specific markers such as *Foxj1, Cdhr4, Pltp, Tm4sf1, Pcp4l1, Tmem212, Stoml3 and Hdc* (**Figure S4** and **Table S5**). These markers were associated with the GO terms glycolipid transport, positive regulation of cholesterol efflux, cilium, and negative regulation of mitotic cell cycle. In contrast, before E16 and along the trajectory from NPCs, we observed the expression of the immature markers *Taf10*, *Kldhc2*, *Tead2*, *Nusap1*, and *Gap43*, associated with cell division, radial glial cell differentiation, and embryonic development. Altogether, these results demonstrate that tanycyte-like cells originate from NPCs early during development and mature starting E16.

## Discussion

This study shows that neurons, typical ependymal cells, and tanycytes arise at different generation rhythms during embryonic development. Neurons mainly arise at E11-13, typical ependymal cells at E13, and tanycytes display a wider developmental generation from E12-P3 without a clear peak when the different subtypes are not considered. Neuron birthdates differ in time and space in the mediobasal hypothalamus, defining a lateral-to-medial gradient for the VMH and a slight rostral-to-caudal gradient for the ARH and DMH. Furthermore, the ependyma arises with an outside-in dorsoventral gradient (*i.e*., typical ependymal cells and β tanycytes are generated before α tanycytes), as well as a marked rostral-to-caudal gradient for α1 et α2 tanycyte populations.

During embryonic development, neural cells originate from radial glial cells (37, 38), which display numerous cell functions such as (1) neurogenic (13, 37, 39) and gliogenic (40–42) competences, (2) helping migration of newborn neurons, and (3) monitoring gyrification of the cerebral cortex (43). First, in agreement with our results, it has been largely described that neurogenesis occurs from E10 to E16 in rodents, peaking between E12 and E14 in the mediobasal hypothalamic regions (8, 19, 24, 44, 45). Basically, it is usually established that hypothalamic neurogenesis peaks at E12-14 in mice and E13-15 in rats (46). Additionally, numerous studies −using the BrdU approach or others− observed a lateral-to-medial gradient in neuron generation that first occurs in the lateral hypothalamus around E11 and finish in the medial hypothalamic nucleus at E13 (8, 44, 45), although some did not find any variations in the timing and the neuroanatomical distribution (47). Here, we confirmed a lateral-to-medial gradient in the generation of the VMH. Additionally, our results showed a slight rostral-to-caudal gradient for neuronal generation in the ARH and DMH. In agreement with these results, such a gradient has already been described (19, 45). Notably, a study demonstrated that neuron birthdate first occurs at E11 in mid-rostral and ventral regions, followed by caudal regions at E14 (20). The authors found that mid-rostral neurons were mainly growth-releasing hormone (GRH)-neurons in the ARH region in rats. Such neuroanatomical generative gradients participate in hypothalamic regionalization and functions (26). Finally, our study does not reveal postnatal neurogenesis while it was already reported in the mediobasal hypothalamus (4). The difference is likely due to the approach we used (*i.e.*, single BrdU injection) that does not allow the visualization of low-rate neurogenesis.

In mice and rats, typical ependymal cells and tanycytes also originate from radial glial cells (13, 43). In the rat, typical ependymal cells are mainly generated from E16 to E18, whereas tanycytes are generated from the last two days of gestation and during the first postnatal week (19). Besides, ependymal cell birthdate precedes tanycyte generation (19, 21, 22), and α2 tanycytes precede α1 tanycyte development (13). Similar developmental birthdates were found in primates where tanycyte differentiation occurs mid-gestation (23). Our results demonstrate a similar chronology with the generation of ependymal cells, followed by β tanycytes and α tanycytes. However, the ependyma generation appears early in mice, starting at E12. Additionally, our data reveal a robust rostral-to-caudal developmental gradient for α tanycytes, with more prominent activity in the caudal region. Altman and Bayer also defined the latest tanycyte generation in the caudal region, extending up to P8 in rats (19). These developmental gradients are also consistent with the fact that, among tanycyte subpopulations, the most dorsal α tanycytes are the last to reach maturity (48). Alternatively, it is worth noticing that a slight caudal-to-rostral gradient is present in the generation of β2 and ventral β1 tanycytes, suggesting a different developmental pattern in the median eminence, but without additional clues in the literature. Finally, comparing tanycyte subtype generative activity over the whole period revealed that α tanycyte generation is more prominent than β subpopulations. Interestingly, α tanycytes are the main subtype maintaining neurogenic competence in the postnatal brain (4, 29) and adulthood (49, 50). Therefore, BrdU incorporation in this subpopulation up to P8 may indirectly be linked to their stem cell properties in postnatal brains. Finally, we propose here the existence of a developmental “outside-in” dorsoventral gradient of generation, by which cells situated in the top (*i.e.*, typical ependymal cells) and bottom of the ventricle (*i.e.*, β tanycytes) generate the first together, followed by cells in the central regions (*i.e.*, α tanycytes). Nothing to our knowledge was previously described in the literature regarding such an unusual dorsoventral gradient along the third ventricle. However, it may also rely on the limited area we analyzed (*i.e.*, the mediobasal hypothalamus) and the way we cut the brain tissue (*i.e.*, 2d coronal sections). Further studies are needed to reconstruct the genesis of the whole third ventricle and obtain a tridimensional picture of ependymal generation gradients.

To understand the temporal and spatial heterogeneity in the generation of the mediobasal 3V ependyma, we used a publicly available molecular atlas of the developing mouse hypothalamus, generated using diencephalic and hypothalamic explants across embryonic and postnatal time points (26). In this study, the authors identified the transcriptional divergence between tanycytes and typical ependymal cells at E13, with the differential expression of cell-type-specific markers such as *Rax* and *Foxj1*, respectively. Consistently, our BrdU analysis highlighted the peak of typical ependymal cell generation at E13. Using the same scRNAseq dataset, we also identified the appearance of tanycyte-like (*Rax*-positive) clusters starting at E14: however, a mature transcriptional profile is more clearly observed during the postnatal developmental time points P4-14, as reported by others (4). Indeed, between P4 and P14, tanycytes increase the expression of numerous markers described in adult cells, such as glucose (*i.e.*, *Slc2a1*, *Slc2a2*) and glutamatergic transporters (*i.e.*, *Slc1a2*, *Slc1a3*) (51–53) and growth factor receptors (*i.e.*, *Fgfr1*, *Igf1*) (54–59). These observations suggest that tanycytes are mature enough between P4 and P14 to respond to variations in energy availability. Using a different approach, Mirzadeh et al. (60) estimated the maturation of tanycytes later by examining the postnatal expression of Nestin, a marker for radial glia (61), and GFAP. Over the first ten days postnatally, the authors observed a decrease in nestin expression and an increase in GFAP expression (60), suggesting that tanycyte maturation continues up to P10. Consistently, morphological and histological studies in the rat and mice have demonstrated that tanycytes begin to fully mature during the first month of life (19, 48, 62). Further studies integrating additional time points during postnatal development would be helpful to expand our knowledge about tanycytes maturation.

The molecular atlas of the developing mouse hypothalamus generated by Kim et al. also allowed us to infer the transcriptional trajectory of ependymal cells during development. Within the tanycyte developmental pseudotime trajectory, we identified E16 as another critical time point for the differentiation of tanycytes. At this point, the expression of specific genes increases, particularly *Nfia*, *Nfib*, and *Nfix*, known as negative regulators of neurogenesis in tanycyte populations (4). Indeed, a study showed recently that tanycyte-specific disruption of the NFI family of transcription factors robustly stimulates tanycyte dedifferentiation, proliferation, and neurogenesis (29). Interestingly, regarding our BrdU analysis, this increased expression of negative regulators of neurogenesis at E16 is concomitant with the end of the neuronal birth and the burst of α tanycytes generation in the mediobasal hypothalamus. Other transcription factors (*i.e.*, *Hes1, Notch2, Egr1, Id4*) and genes (*i.e.*, *Apoe, Cd63, Cnih2, Arxes2*) have also been identified during tanycyte differentiation (starting E16) and maturation (starting P4), and constitute excellent candidates for controlling the specification of these peculiar hypothalamic cell types. Interestingly, the helix-loop-helix gene *Hes1*, together with *Notch1*, responds to *Rax* to promote Muller glia cell differentiation (63). Further exploration of these candidates is needed to understand their role in tanycyte development better.

In conclusion, the ontogeny of ependymoglial cells diverges according to cell type, time, and neuroanatomical distribution. Our original cellular and molecular approach allowed us to demonstrate that tanycytes originate around E13, start to differentiate as early as E16, and reach full maturation during early postnatal development (P4-14). Globally, our data allowed us to generate a comprehensive spatiotemporal atlas of tanycyte birth and development and identify molecular candidates involved in the development of tanycytes.

## Data availability statement

The datasets presented in this study can be found in online repositories. The names of the repository/repositories and accession number(s) can be found in the article/**Supplementary Material**.

## Ethics statement

The animal study was reviewed and approved by Veterinary Office of Canton de Vaud & University of Lausanne.

## Author contributions

DL-R contributed to data analysis, performed the bioinformatic analysis and wrote the manuscript. AR, ML and MG performed the experiments and participated in data analysis. SC and FL contributed to conception and design of the study, performed experiments, participated in data analysis and wrote the manuscript. All authors contributed to the article and approved the submitted version.
