# Supplementary material for: Ontogeny of ependymoglial cells lining the third ventricle in mice
Source: Front Endocrinol (Lausanne). 2023 Jan 5;13:1073759. doi: 10.3389/fendo.2022.1073759 (PMC9849764; doi:10.3389/fendo.2022.1073759)
Supplement: Supplementary file 6 [file DataSheet_1.pdf]

Supplementary Material

Ontogeny of ependymoglial cells lining the third ventricle in mice.

Authors: Lopez-Rodriguez D[1], Rohrbach A[1,3], Lanzillo M[2,3], Gervais M[2], Croizier S[2,4], Langlet F[1,4].

Affiliations:

[1] Department of Biomedical Sciences, Faculty of Biology and Medicine, University of Lausanne, Lausanne, Switzerland.

[2] Center for Integrative Genomics, Faculty of Biology and Medicine, University of Lausanne, Lausanne, Switzerland

[3] Co-second author

[4] Co-last author

Corresponding author:

Langlet, Fanny (fanny.langlet@unil.ch)

Department of Biomedical Sciences,

University of Lausanne,

Bugnon 7, 1005 Lausanne

Switzerland.

Supplementary Material – Ontogeny of ependymoglial cells lining the third ventricle in mice

List of tables

Table S1. List of primary antibodies used for immunohistochemistry (see excel file).

Table S2. List of secondary antibodies used for immunohistochemistry (see excel file).

Table S3. Quantification for double-stained BrdU/NeuN and BrdU/Vimentin-positive cells (see excel file).

Table S4. Features and gene ontology terms expressed in tanycyte-like clusters across developmental time points (see excel file).

Table S5. Developmental dynamics in gene expression in the transition from NPCs to tanycyte-like cells and ependyma (see excel file).

List of figures

Figure S1. Developmental hypothalamic BrdU incorporation per zone in NeuN-positive cells.

Figure S2. Developmental hypothalamic BrdU incorporation per zone in Vim-positive cells.

Figure S3. scRNAseq analysis of hypothalamic Rax-positive cells.

Figure S4. Developmental pseudotime trajectory of ependymal cells from NPCs.

List of documents

File S1. Raw pictures showing BrdU labeling in zones 1, 2, 3, and 4 from «E10» to «E18» brains.

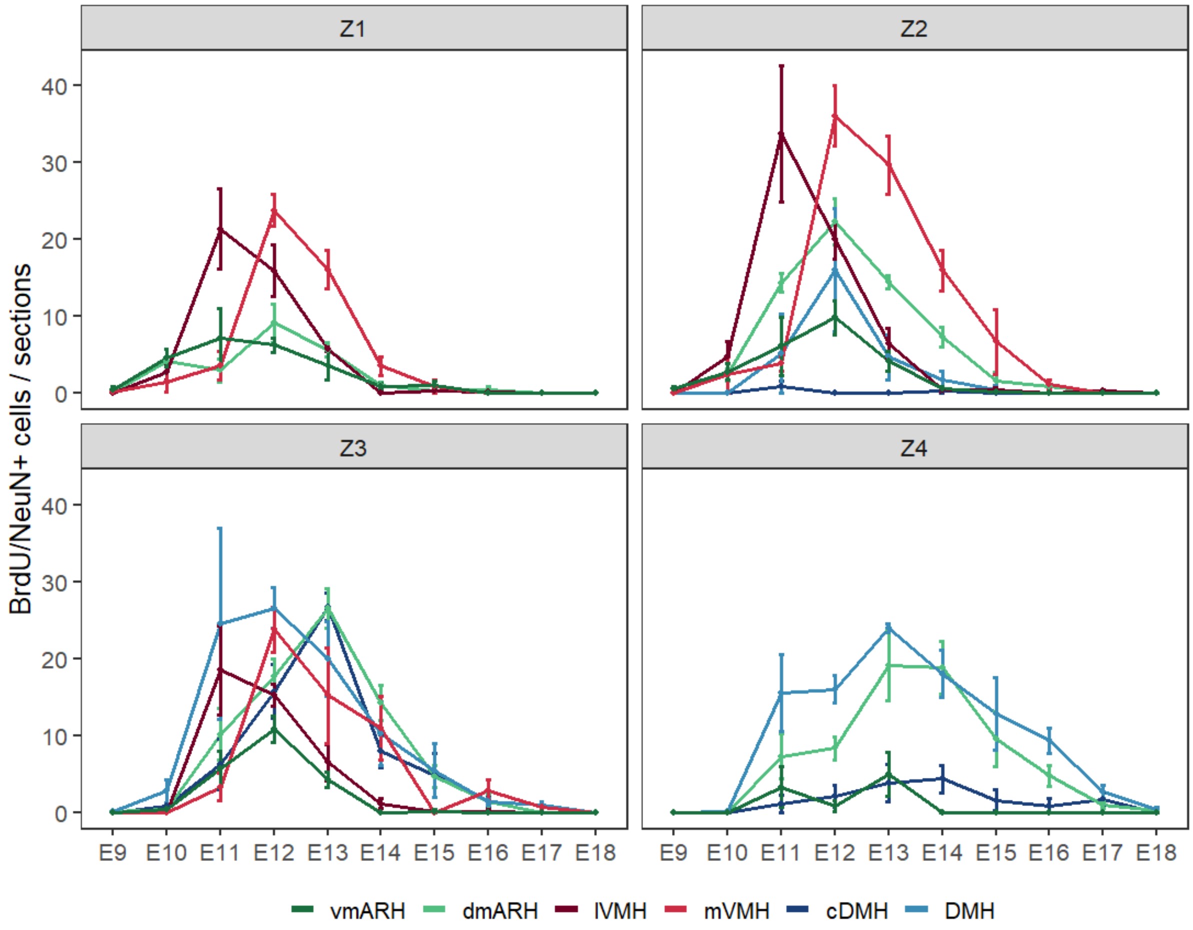

Figure S1. Developmental hypothalamic BrdU incorporation per zone in NeuN-positive cells. Number of BrdU/NeuN-positive cells per hypothalamic region and per age in each rostrocaudal zone (1 to 4). "E9" brains were harvested from P21-22 male pups whose mothers received a single BrdU injection during pregnancy at E9; "E10" brains were harvested from P21-22 male pups whose mothers received a single BrdU injection during pregnancy at E10... cDMH: compact dorsomedial nucleus of the hypothalamus, dmARH: dorsomedial arcuate nucleus of the hypothalamus, DMH: dorsomedial nucleus of the hypothalamus, lVMH: lateral ventromedial nucleus of the hypothalamus, mVMH: medial ventromedial nucleus of the hypothalamus, vmARH: ventromedial arcuate nucleus of the hypothalamus.

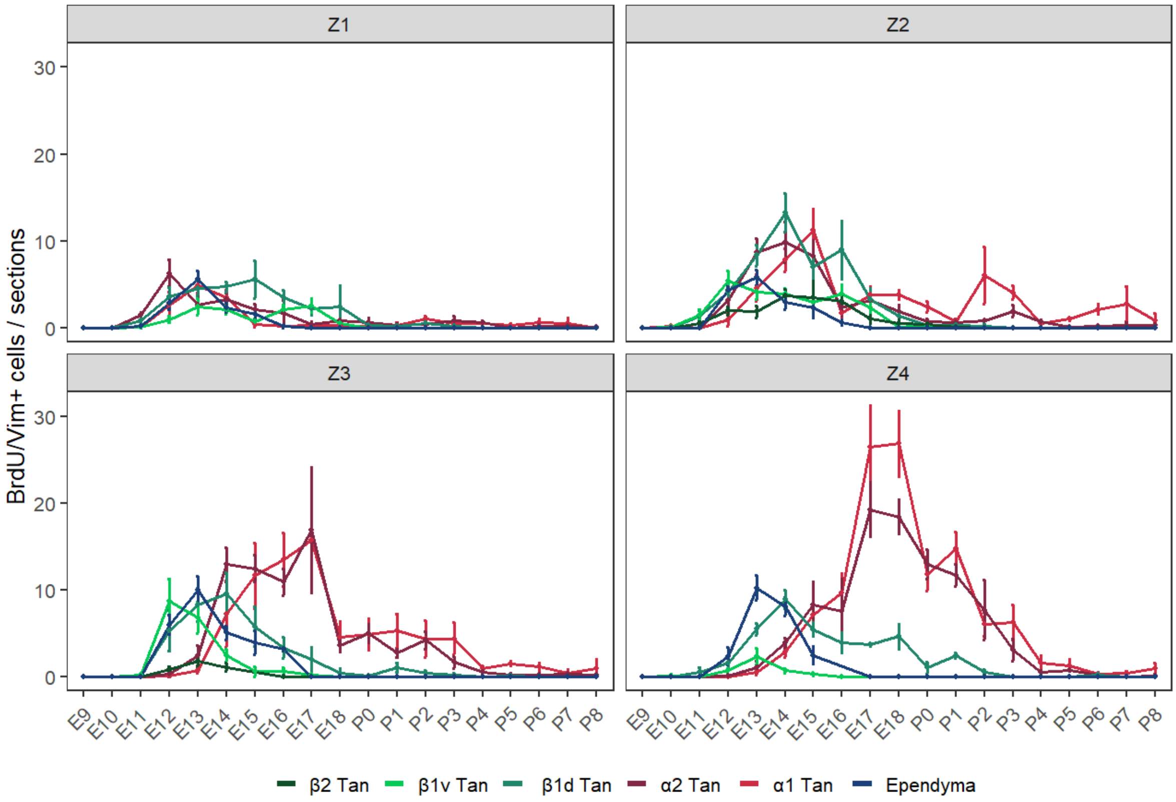

Figure S2. Developmental hypothalamic BrdU incorporation per zone in Vim-positive cells. Number of BrdU/Vim-positive cells per ependymal subpopulation and per age in each rostrocaudal zones (1 to 4). "E9" brains were harvested from P21-22 male pups whose mothers received a single BrdU injection during pregnancy at E9; "E10" brains were harvested from P21-22 male pups whose mothers received a single BrdU injection during pregnancy at E10… α1 Tan: alpha1 tanycyte, α2 Tan: alpha2 tanycyte, β1v Tan: ventral beta1 tanycyte, β1d Tan: dorsal beta1 tanycyte, and β2 Tan, beta2 tanycytes.

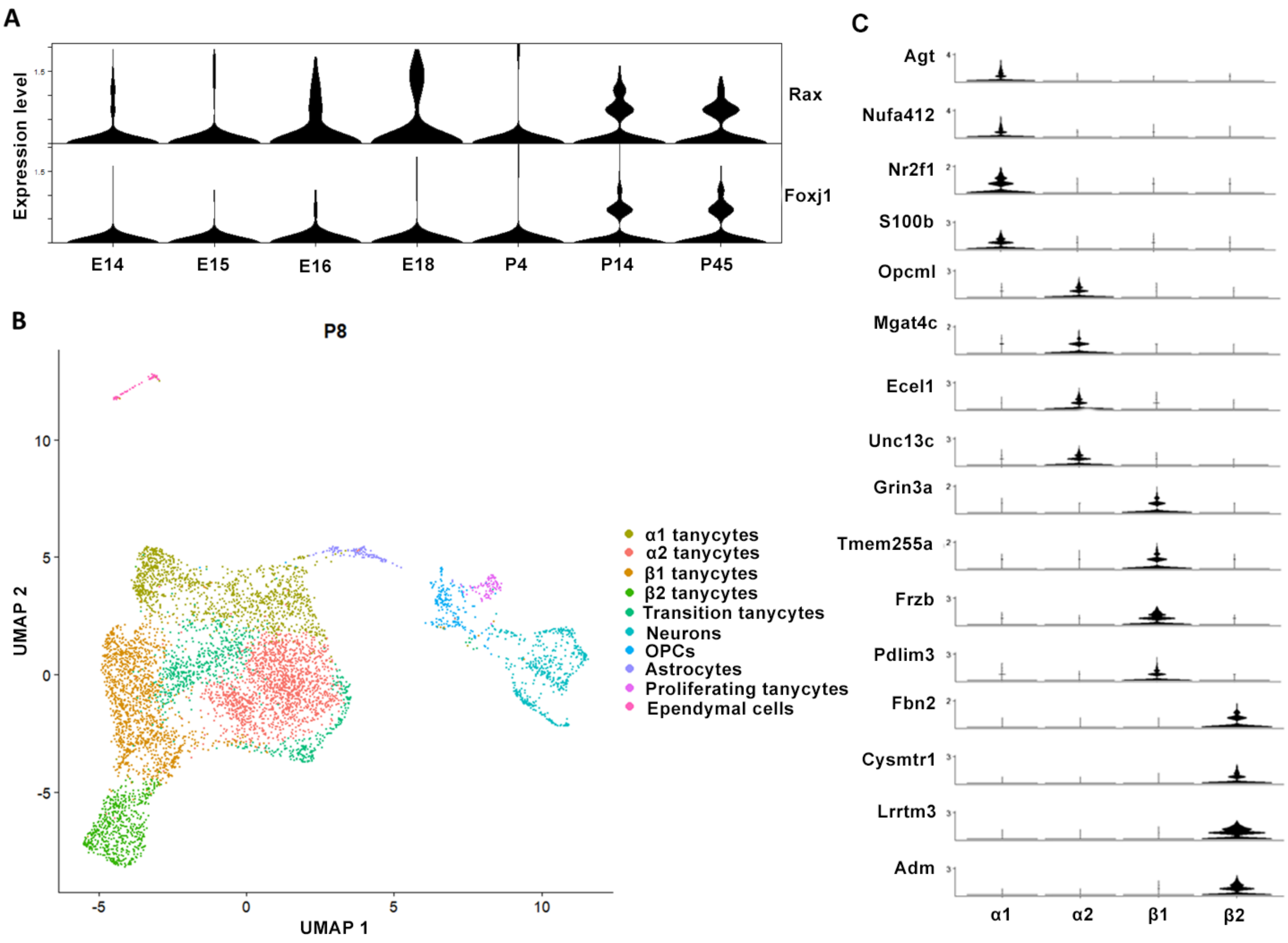

Figure S3. scRNAseq analysis of hypothalamic Rax-positive cells. **A** Violin plot showing the expression of *Rax* and *Foxj1* in ependymoglial cells across development. Data from Kim et al. (2020). **B** UMAP plot of scRNAseq data from control WT mice at P8. Neurons, ependymal and tanycyte subtype clusters are displayed by color shading. Data from Yoo et al. (2021). **C** Violin plot of the tanycyte specific alpha1, alpha2, beta1 and beta2 subtypes markers identified in the dataset. Data from Yoo et al. (2021).

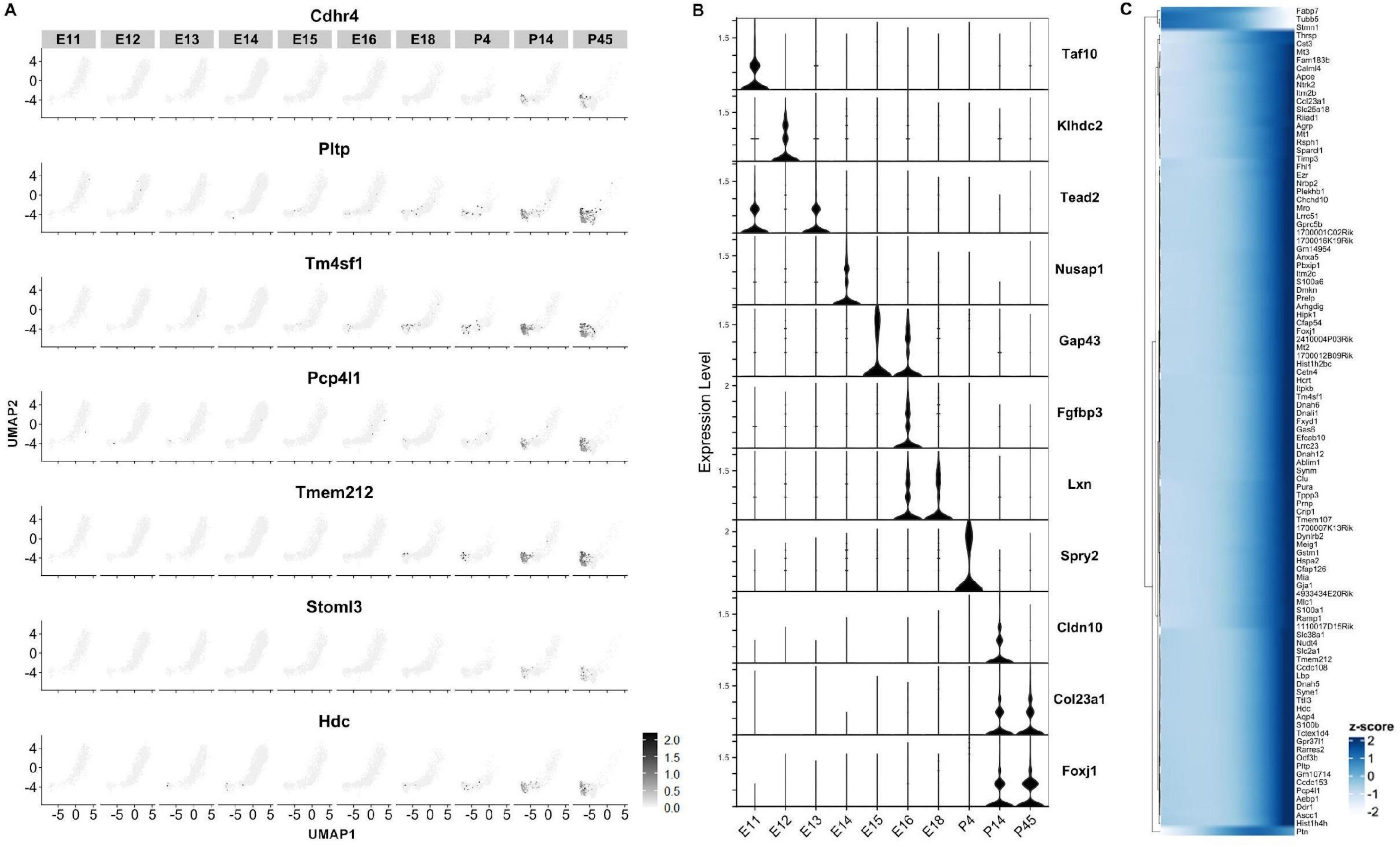

Figure S4. Developmental pseudotime trajectory of ependymal cells from NPCs. **A** UMAP plot splitted by developmetal time point showing the expression of features specifically expressed in the ependyma. **B** Violin plot of markers specifically expressed across the different developmetal time points of the scRNAseq dataset. **C** Heatmap showing the developmental trajectory of the ependyma cell population from NPCs. Data from Kim et al. (2020).

File S1. Raw pictures showing BrdU labeling in zones 1, 2, 3, and 4 from «E10» to «E18» brains. A differential pattern of BrdU labeling can be observed: on one side, BrdU labeling does not fill out the entire nucleus of the cell, displaying a “diffuse” labeling pattern. On another side, BrdU staining filled out the entire cell nucleus, displaying a “full” labeling pattern. Indications regarding these labelling are given for the main ependymal subpopulations, at each time point, for each rostrocaudal zone. NA, not applicable. For the rostrocaudal and dorsoventral subdivision, see figure 1.

« E10 » brains: BrdU injection at E10 => Sacrifice at P21

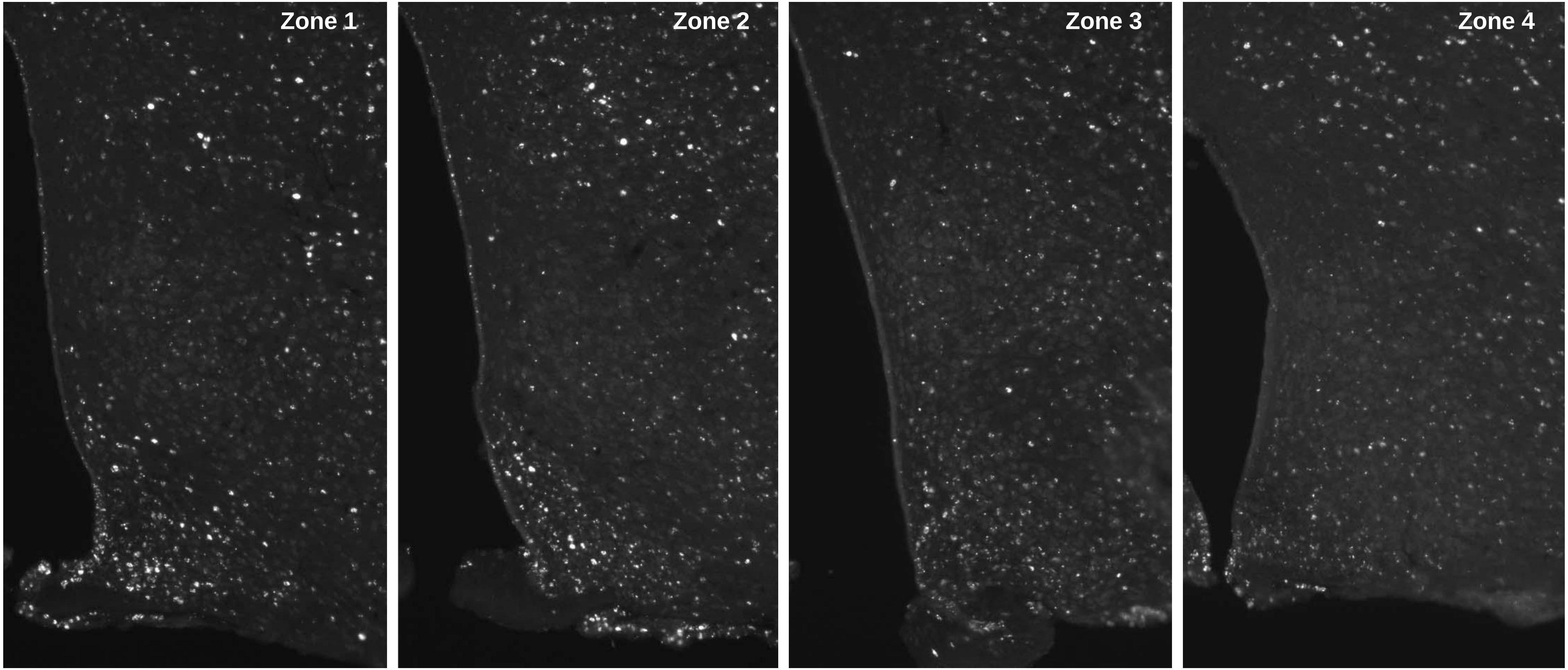

	Zone 1	Zone 2	Zone 3	Zone 4
**Ependyma**	NA	NA	NA	NA
**Alpha1**	NA	NA	NA	NA
**Alpha2**	NA	NA	NA	NA
**Beta1**	Diffuse	Diffuse	Diffuse	Diffuse

« E11 » brains: BrdU injection at E11 => Sacrifice at P21

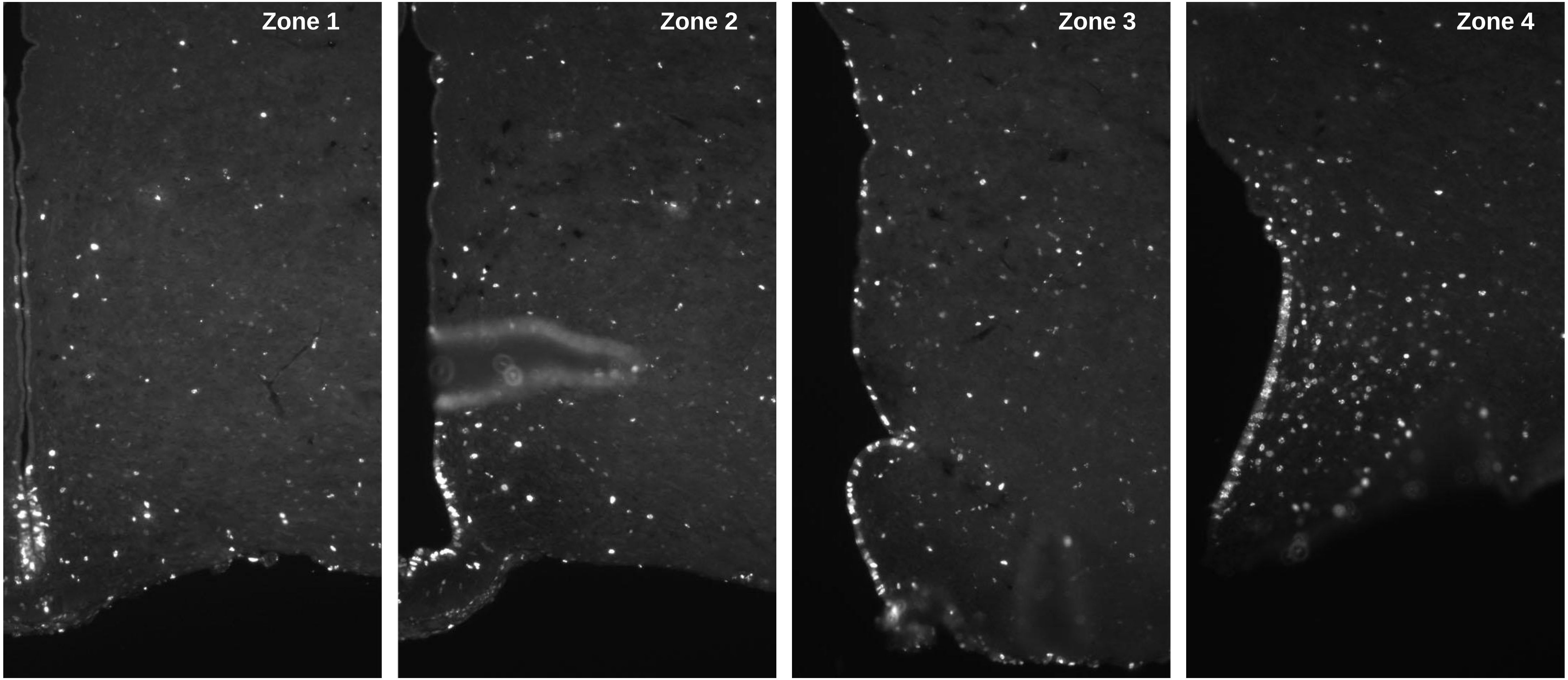

	Zone 1	Zone 2	Zone 3	Zone 4
**Ependyma**	NA	NA	NA	NA
**Alpha1**	NA	NA	NA	Diffuse
**Alpha2**	NA	NA	NA	Diffuse
**Beta1**	Full	Full	Diffuse	Diffuse

« E12 » brains: BrdU injection at E12 => Sacrifice at P21

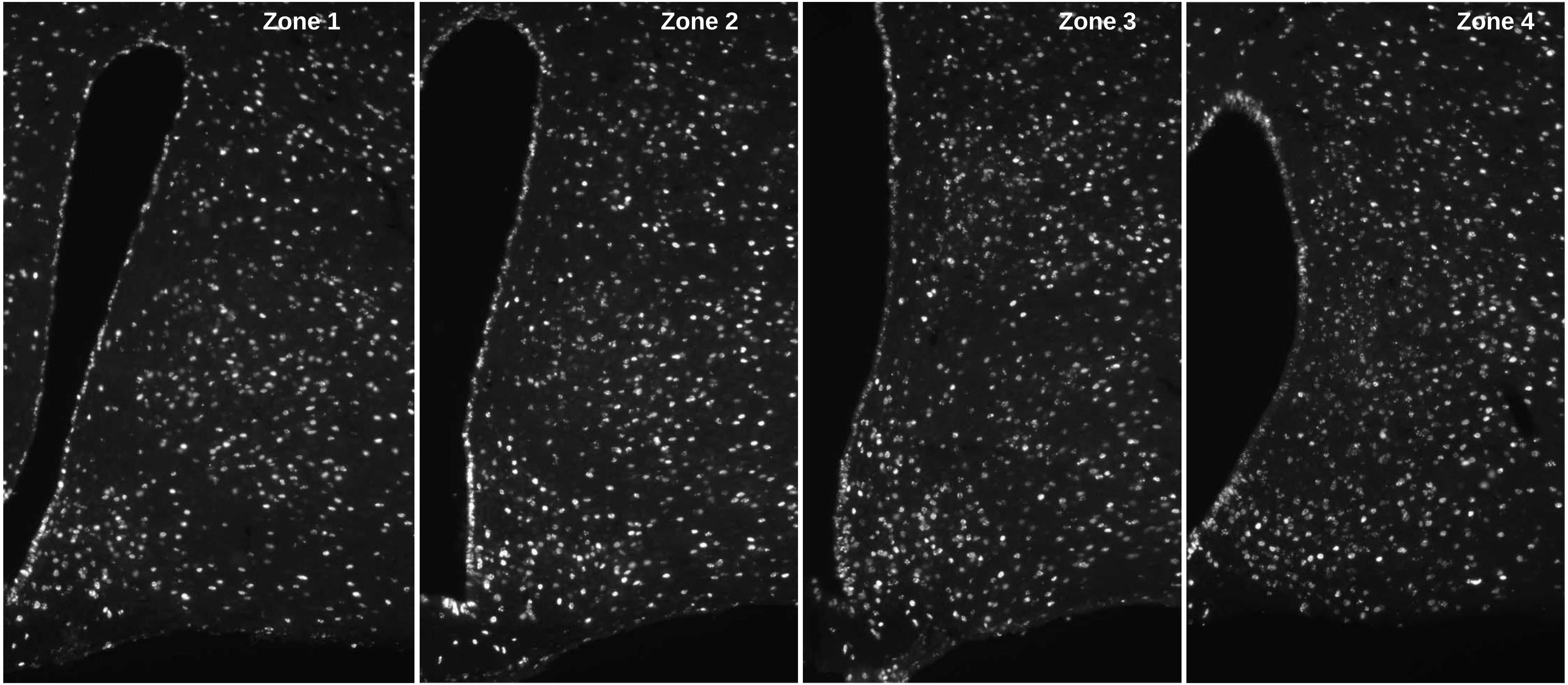

**Ependyma**	Diffuse/Full	Diffuse/Full	Diffuse/Full	Diffuse
**Alpha1**	Full	Diffuse	Diffuse	Diffuse
**Alpha2**	Full	Full	Diffuse	Diffuse
**Beta1**	Full	Full	Diffuse	Diffuse

« E13 » brains: BrdU injection at E13 => Sacrifice at P21

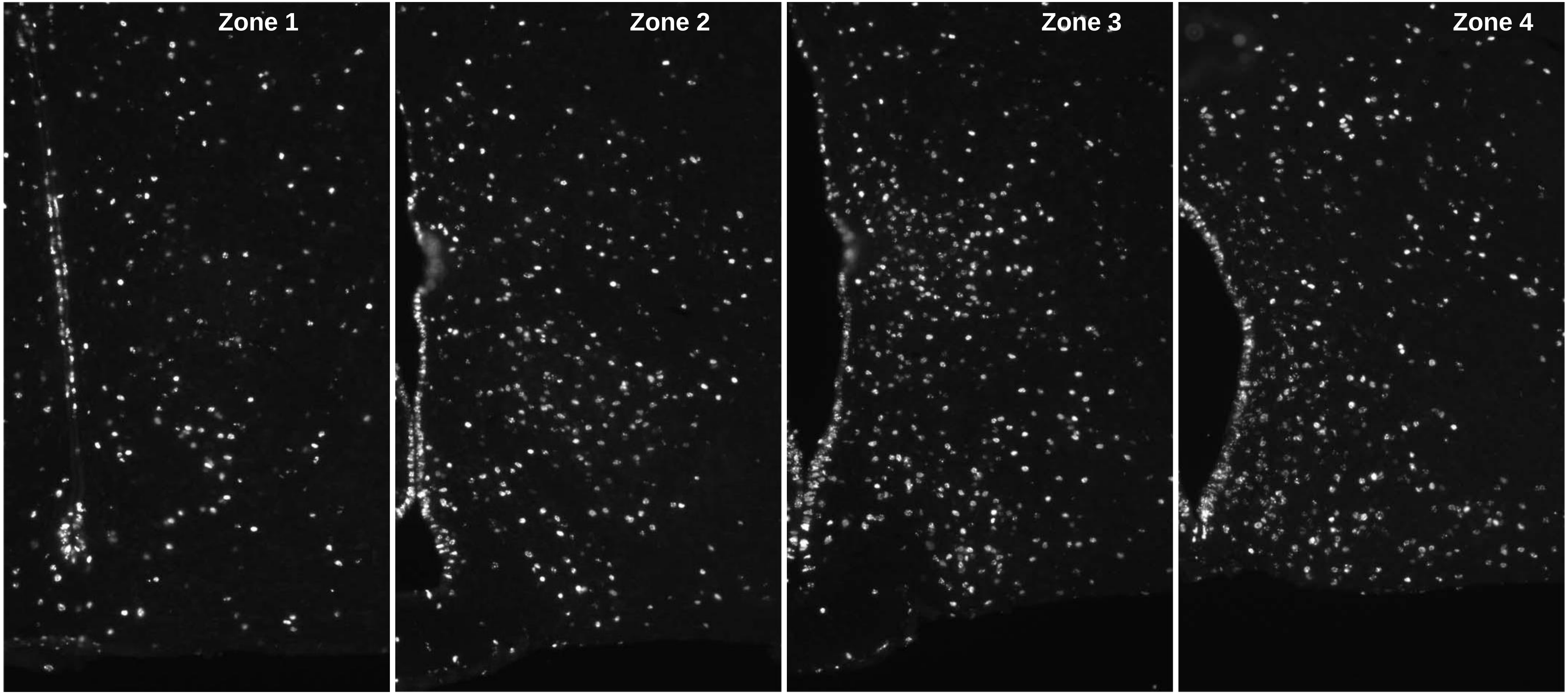

**Ependyma**	Full	Full	Full	Full
**Alpha1**	Full	Diffuse/Full	Diffuse	Diffuse
**Alpha2**	NA	Full	Diffuse	Diffuse
**Beta1**	Full	Full	Full	Full

« E14 » brains: BrdU injection at E14 => Sacrifice at P21

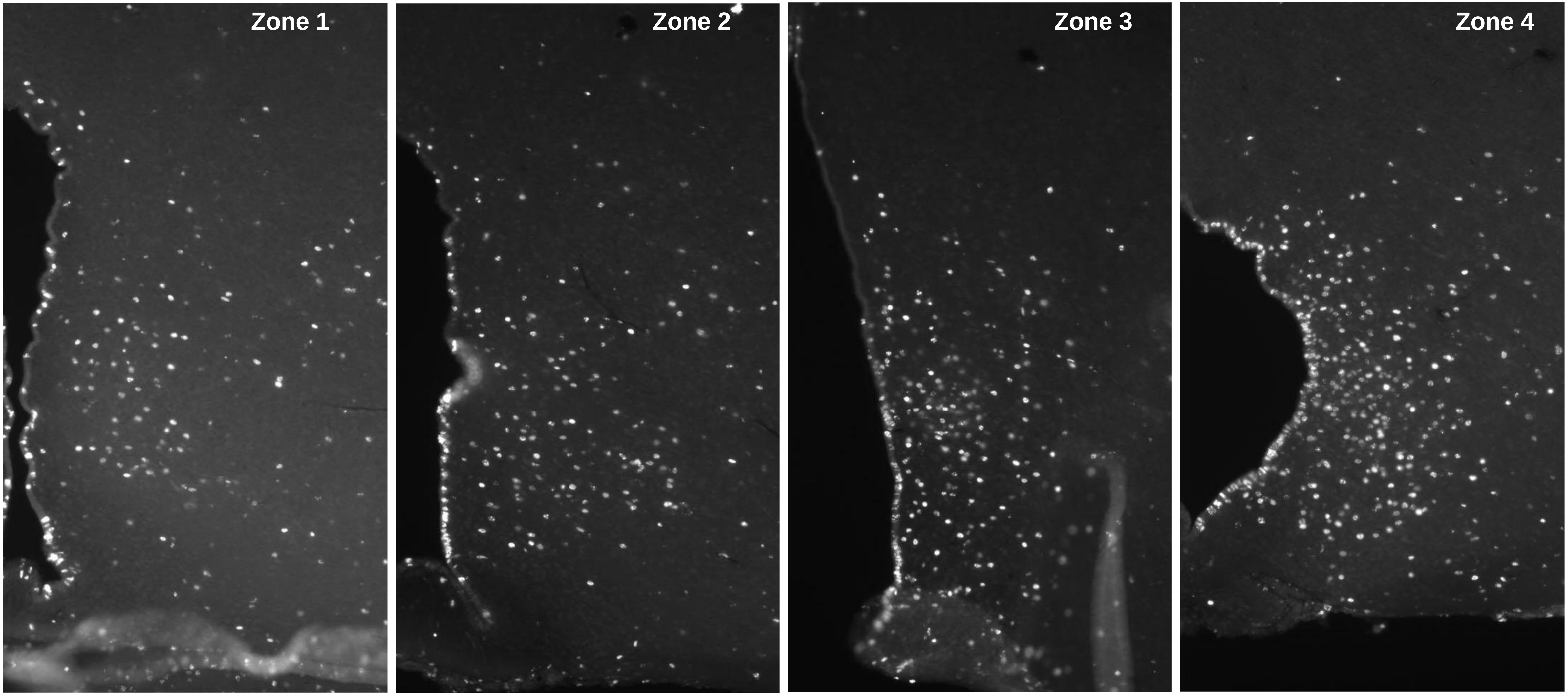

	Zone 1	Zone 2	Zone 3	Zone 4
**Ependyma**	NA	NA	NA	NA
**Alpha1**	NA	**Full**	**Diffuse**	**Diffuse**
**Alpha2**	NA	**Full**	**Full**	**Diffuse**
**Beta1**	**Full**	NA	ND	**Full**

« E15 » brains: BrdU injection at E15 => Sacrifice at P21

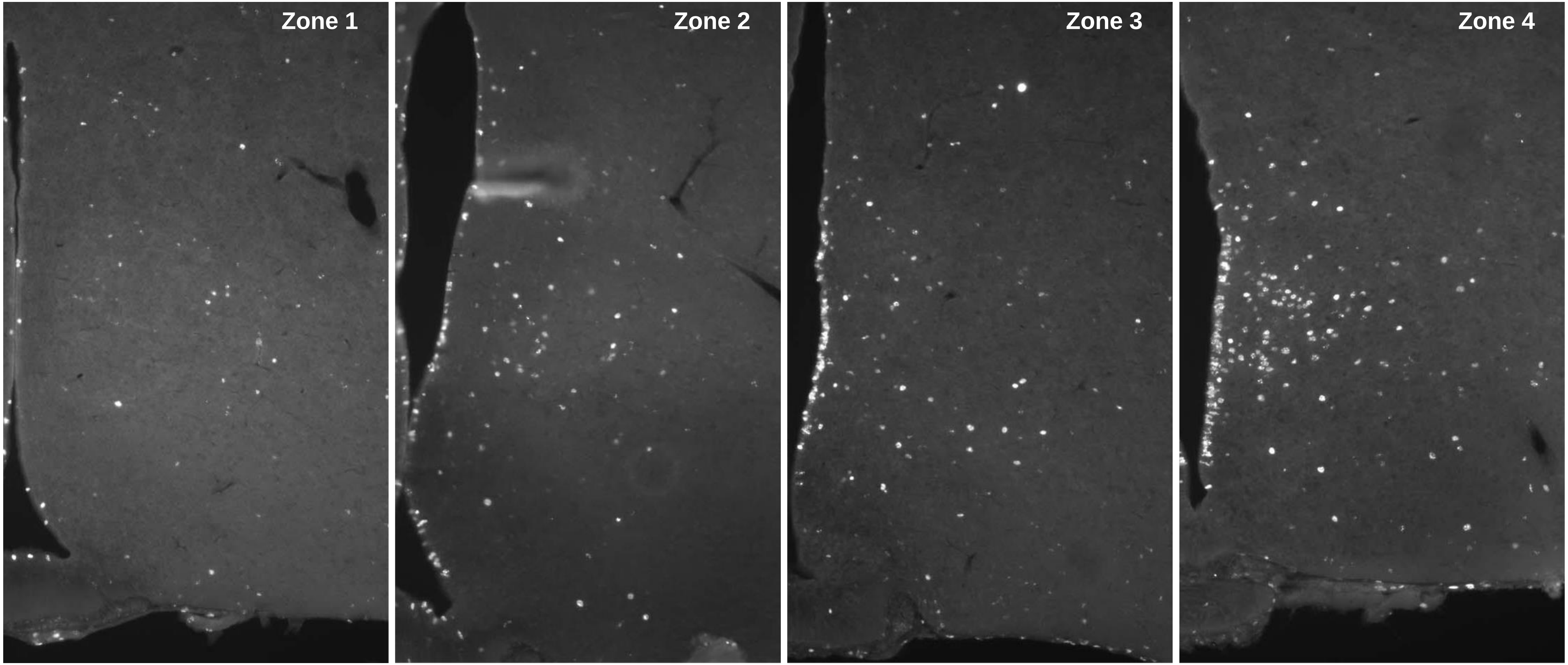

**Ependyma**	NA	NA	NA	NA
**Alpha1**	NA	**Full**	**Diffuse**	**Diffuse**
**Alpha2**	NA	NA	**Full**	**Diffuse**
**Beta1**	NA	NA	NA	NA

« E16 » brains: BrdU injection at E16 => Sacrifice at P21

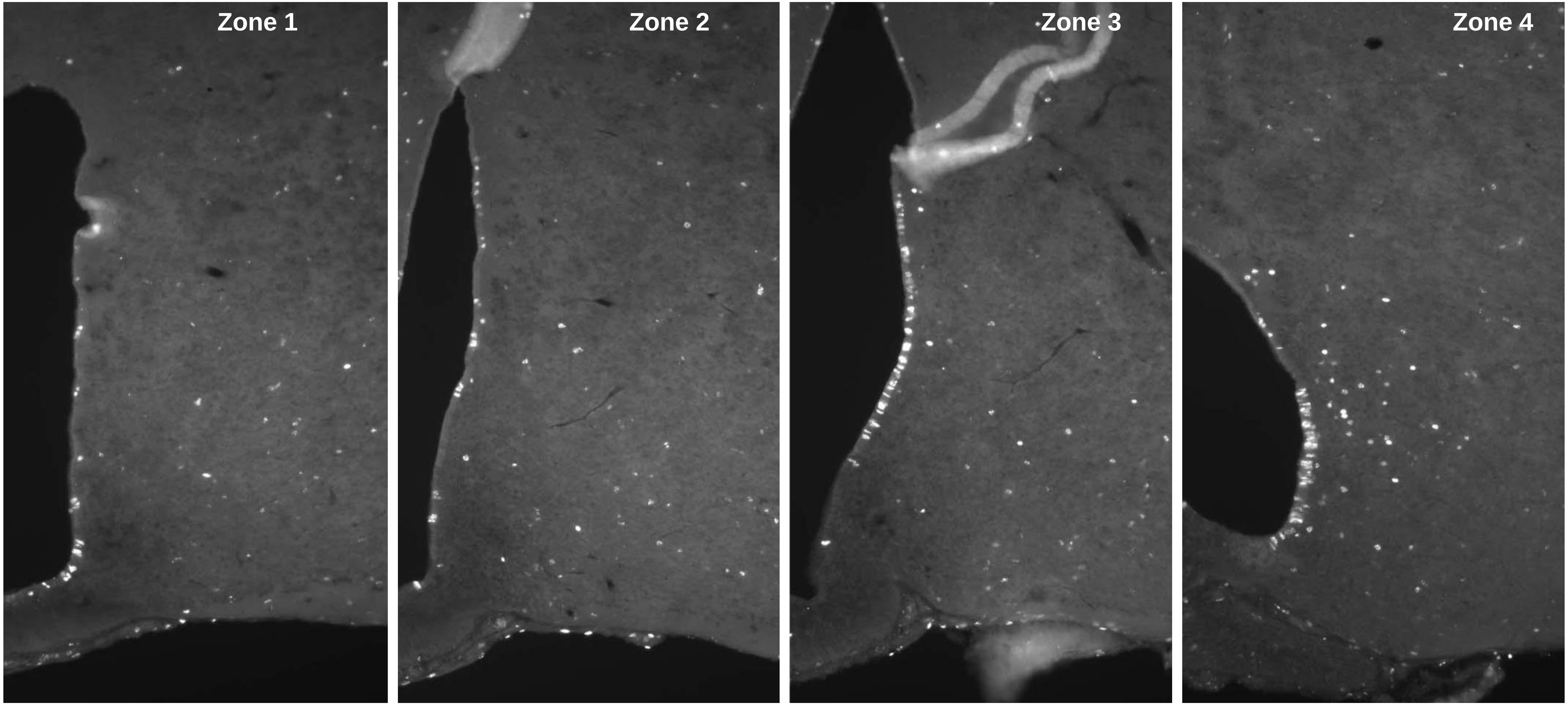

	Zone 1	Zone 2	Zone 3	Zone 4
**Ependyma**	NA	NA	NA	NA
**Alpha1**	NA	NA	**Full**	**Full**
**Alpha2**	NA	NA	**Full**	**Full**
**Beta1**	**Full**	NA	NA	NA

« E17 » brains: BrdU injection at E17 => Sacrifice at P21

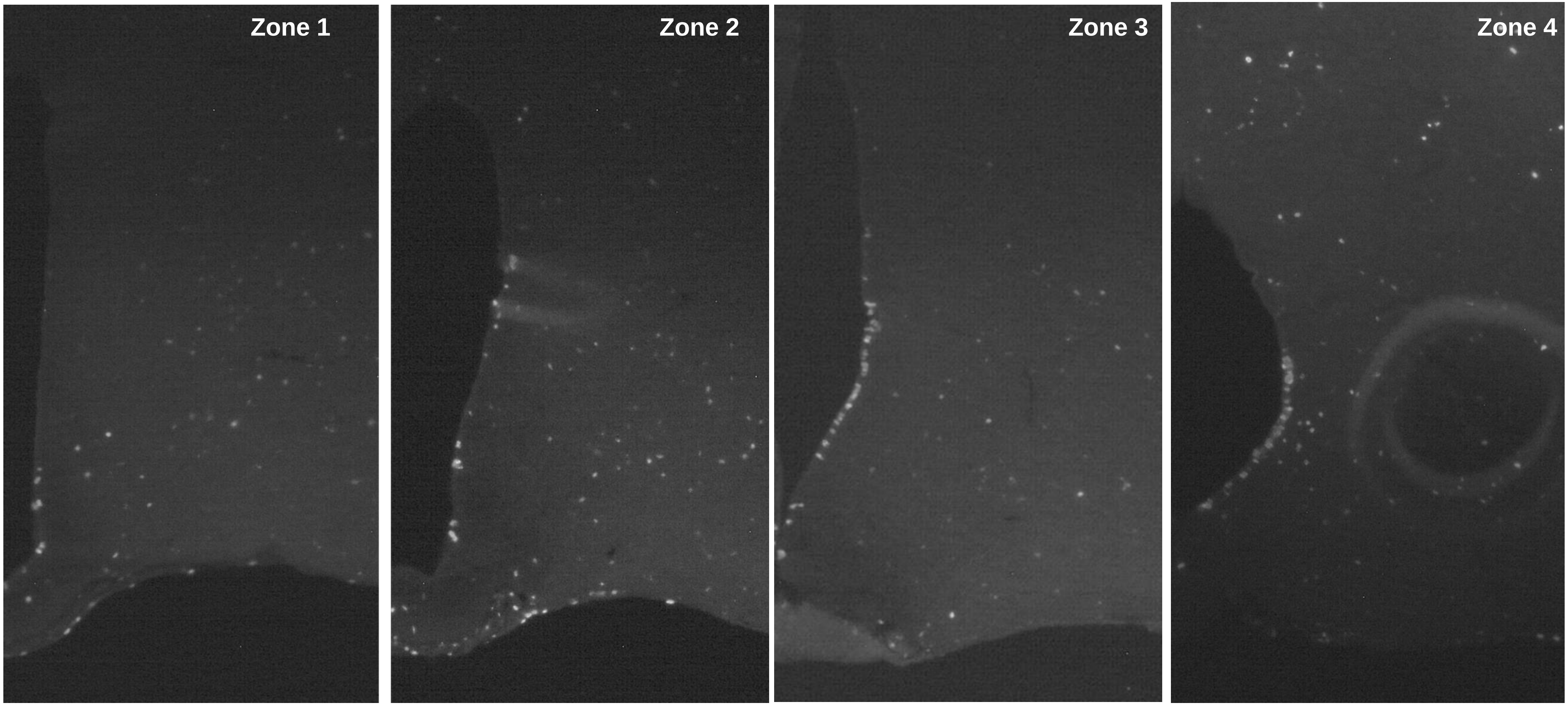

**Ependyma**	NA	NA	NA	NA
**Alpha1**	NA	NA	**Full**	**Full**
**Alpha2**	NA	NA	**Full**	**Full**
**Beta1**	NA	NA	NA	NA

« E18 » brains: BrdU injection at E18 => Sacrifice at P21

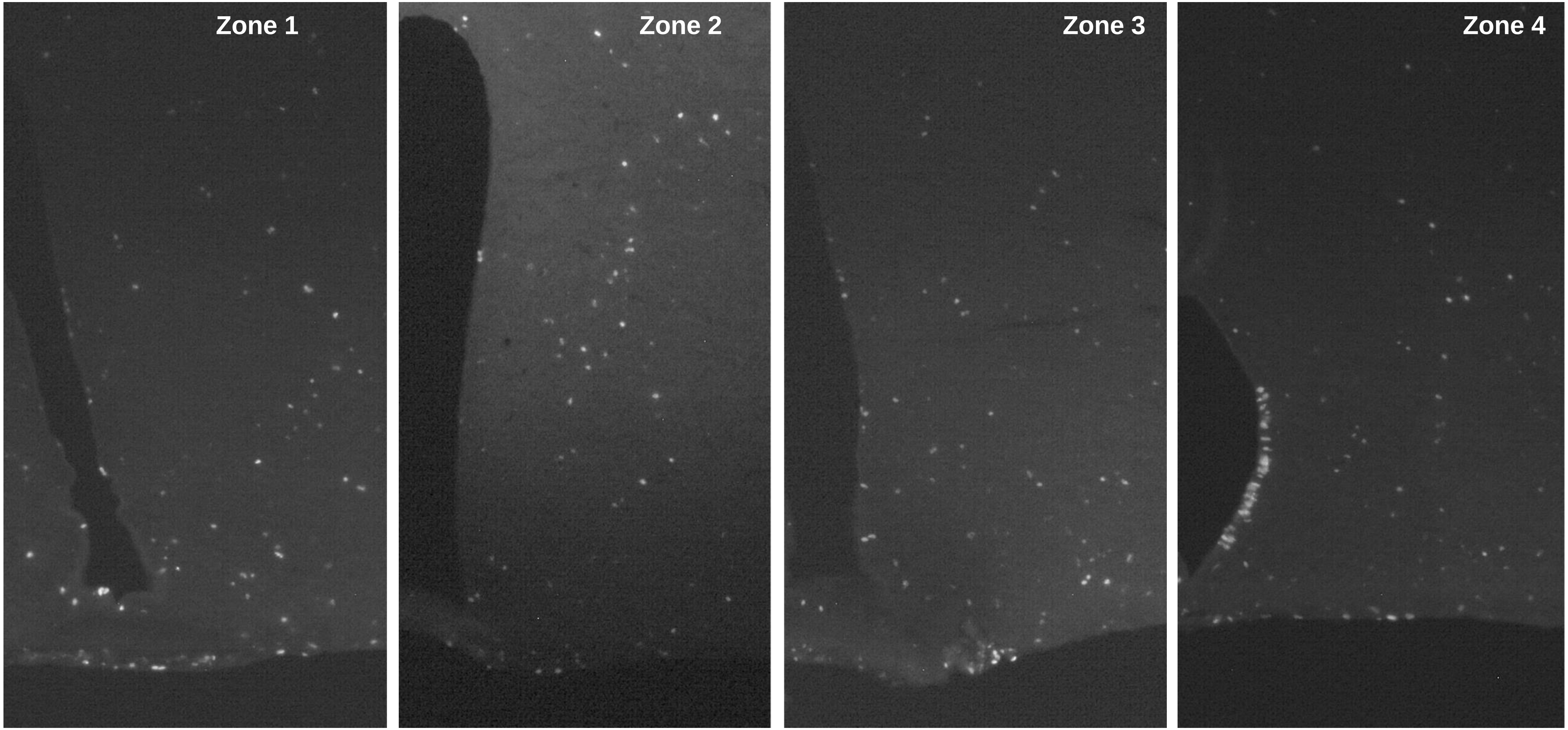

**Ependyma**	NA	NA	NA	NA
**Alpha1**	NA	NA	NA	Full
**Alpha2**	NA	NA	NA	Full
**Beta1**	NA	NA	NA	NA
